# Cancer Specific CAIX‐Targeting Supramolecular Lysosome‐Targeting Chimeras (Supra‐LYTAC) for Targeted Protein Degradation

**DOI:** 10.1002/advs.202503134

**Published:** 2025-04-03

**Authors:** Dohyun Kim, Gyeongseok Yang, Chaelyeong Lim, Gaeun Park, Jaemo Lee, Youjung Sim, Ja‐Hyoung Ryu

**Affiliations:** ^1^ Chemistry Department Ulsan National Institute of Science and Technology (UNIST) 50, UNIST‐gil, Eonyang‐eup, Ulju‐gun Ulsan 44919 Republic of Korea

**Keywords:** carbonic anhydrase IX, lysosome‐targeting chimeras, peptide amphiphile, Supramolecular assembly, targeted protein degradation

## Abstract

Recently, targeted protein degradation (TPD) strategies have emerged as a promising solution to tackle undruggable proteins. While most TPD strategies target intracellular proteins, limited options exist for targeting extracellular or membrane proteins. Herein, cancer specific carbonic anhydrase IX (CAIX)‐targeting supramolecular nanofibrous lysosome‐targeting chimeras (Supra‐LYTAC) is reported. Two self‐assembling amphiphilic peptides are synthesized: one that interacts with the protein of interest (POI), and another that mediates lysosomal endocytosis by targeting a cancer‐specific enzyme. Notably, these two peptides co‐assemble into nanofibers capable of targeting cancer cells in a spatiotemporal manner. Through dynamic and multivalent binding, a ternary complex form (supramolecular chimeric nanostructure; CAIX‐nanofiber‐POI), which undergoes internalization into lysosomes where the POI is degraded through lysosomal catalytic activity. This study demonstrates the potential of supramolecular approaches to expand the scope of LYTAC technology, offering new opportunities for designing TPD strategies in the future.

## Graphical abstract

Spatiotemporal Supra‐LYTAC formation near cancerous membrane via CAIX‐targeting is achieved. As a result of generation of Supra‐LYTAC, POI trafficking into lysosome is significantly implemented for targeted protein degradation.

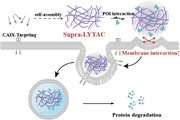

## 1 Introduction

In 2001, proteolysis‐targeting chimeras (PROTAC) has been first proposed in the literature, demonstrating targeted protein degradation (TPD) by recruiting intracellular protein degradation system, specifically the ubiquitin‐proteosome system.^[^ ^1^, ^2^, ^3^, ^4^, ^5^, ^6^, ^7^ ^]^ Unlike classical drugs, PROTACs consist of two heterobifunctional ligands connected by a linker. One ligand binds to protein‐of‐interest (POI), and the other recruits E3 ubiquitin ligase. The simultaneous binding drives the formation of a ternary complex (POI‐chimeric molecule‐E3 ubiquitin ligase) for subsequent degradation. This event‐driven pharmacology has demonstrated potential in addressing even undruggable proteins. The evolution of TPD strategies led to the development of lysosome‐targeting chimeras (LYTAC), which direct POIs to lysosomes for degradation.^[^ ^8^, ^9^ ^]^ Unlike PROTACs that recruit E3 ubiquitin ligase in the cytoplasmic matrix, LYTACs form ternary complexes with POIs and lysosome‐trafficking receptors in the extracellular matrix. This distinction enables LYTACs to address extracellular or membrane‐anchored POIs. Recent years have seen the development of chimeric molecules based on antibodies/aptamers targeting mannose‐6‐phosphate receptor or asialoglycoprotein receptor on the cell membrane as lysosomal‐trafficking receptors, modulating cellular behavior through protein degradation.^[^ ^10^, ^11^, ^12^, ^13^, ^14^, ^15^ ^]^ Additionally, small molecule‐based LYTACs targeting RGD receptor, covalently linked LYTACs, and peptide based LYTACs have been developed for POI degradation.^[^ ^16^, ^17^, ^18^, ^19^, ^20^ ^]^ While TPD strategies have shown promise in degrading undruggable proteins, these technologies are still in their infancy and face challenges including high molecular weight, low cell permeability, off‐target effects, and high costs associated with linker optimization.^[^ ^21^, ^22^ ^]^ These limitations underscore the urgent need for alternative LYTAC strategies.

**Scheme 1:**
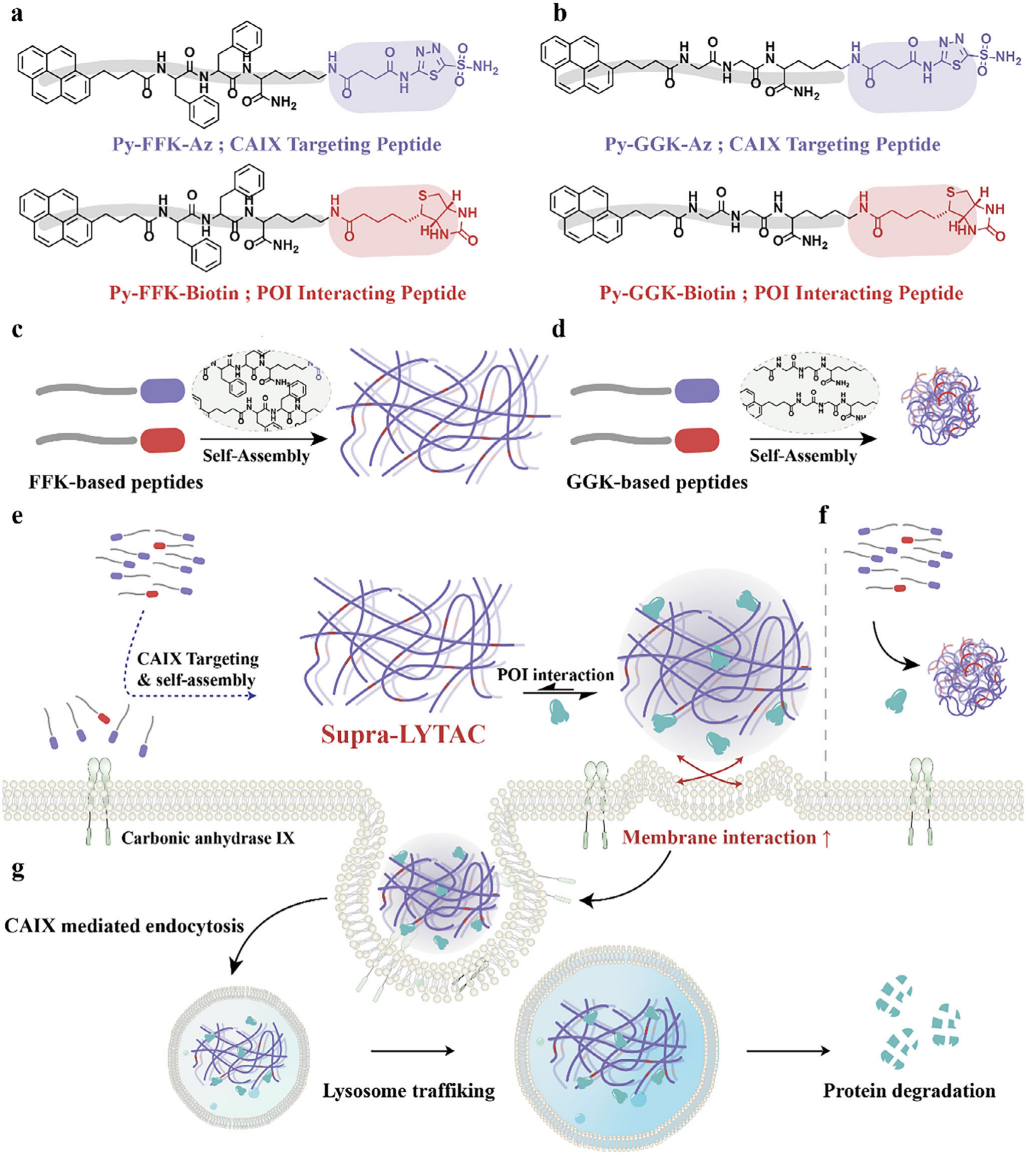
a,b) Monomer design for Supra‐LYTAC; c,d) Illustration demonstrating co‐assembly to yield nanofiber and nanosphere. The nanofiber structure could be constructed in FFK‐based peptides due to the high self‐assembly propensity, while GGK‐based peptides generate nanosphere structure; e) Supra‐LYTAC constructed in the cancerous membrane by targeting CAIX. It can further interact with POI, as followed by internalization into lysosome via CAIX‐induced endocytosis; f) Nanosphere showing weak ability for the internalization into lysosome due to low self‐assembly propensity; g) POI internalizing into lysosome with Supra‐LYTAC could be degraded in the lysosome.

In previous decades, supramolecular self‐assembly has been emerged as an effective tool for developing novel functional nano‐platforms^[^ ^23^, ^24^, ^25^, ^26^, ^27^, ^28^, ^29^, ^30^, ^31^ ^]^ due to its dynamic properties and multivalent binding characteristics. Recently, supramolecular PROTAC have gained significant attention as an alternative strategy beyond conventional small molecule‐based or antibody/aptamer‐based PROTACs.^[^ ^32^, ^33^, ^34^, ^35^ ^]^ In addition, supramolecular LYTAC has also gained significant attention for the past two years, as demonstrated in recent literature including liposome, nanoparticle, and nucleic acid based LYTAC.^[^ ^36^, ^37^, ^38^, ^39^, ^40^, ^41^, ^42^, ^43^, ^44^, ^45^ ^]^ While the great achievement for supramolecular LYTAC in TPD field, spatiotemporal control for chimeric structure in extracellular domain remained a challenge. Our previous study demonstrated extracellular nanofiber formation spatiotemporally via CAIX‐targeting.^[^ ^46^ ^]^ Notably, CAIX can mediate endocytosis inside the cells, which has the advantage of internalizing extracellularly formed self‐assemblies into lysosome. Inspired by the supramolecular self‐assembly characteristics and previous work, we proposed a cancer specific CAIX‐targeting supramolecular nanofibrous LYTAC (Supra‐LYTAC). Specifically, we developed two hetero‐functionalized self‐assemble amphiphilic FFK‐based peptides: one targeting carbonic anhydrase IX (CAIX) enzyme expressed on cancerous cell membranes, and another interacting with POI (**Scheme** **1**a). To validate our hypothesis for Supra‐LYTAC, we additionally designed GGK‐based peptide containing weak secondary interaction (Scheme 1b). In the case of FFK‐based peptides, two functional peptides homogeneously co‐assemble into nanofibers due to strong secondary interactions between peptide backbones (Pyrene‐Phe‐Phe) (Scheme 1c). This bifunctional supramolecular nanofiber can be specifically constructed in cancerous cell membranes through a high binding affinity of acetazolamide with CAIX. Simultaneously, the supramolecular nanofiber near CAIX interacts with the designated POI, resulting in ternary complex formation (CAIX‐nanofiber‐POI) (Scheme 1e). These complexes are internalized into lysosomes via clathrin‐mediated endocytosis. The high catalytic lysosomal environment facilitates the degradation of lysosomal‐localized POI (Scheme 1g). In contrast to FFK‐based peptides with high self‐assembly propensity, GGK‐based peptides possess weak secondary interaction (Pyrene‐Gly‐Gly), yielding nanosphere structure.

(Scheme 1d). This weak secondary interaction disadvantageously affects the creation of supramolecular chimeric nanostructures near cellular membranes due to low non‐covalent interaction with POI (Scheme 1f). Thus, supramolecular LYTAC efficiency is significantly mitigated compared to FFK‐based peptides.

## 2 Results and Discussion

### 2.1 Self‐Assembly Propensity of Peptide Amphiphiles and Complex Formation with Protein‐of‐Interest

Supramolecular self‐assembly serves as an important tool for creating functional nanomaterials through dynamic properties and multivalent binding characteristics that cannot be achieved in the monomeric state. Amphiphilic peptides are excellent monomer candidates for biological applications due to their biocompatibility, ease of synthesis, and high engineering potential through peptide library combinations.^[^ ^47^, ^48^, ^49^ ^]^ Among these, the Pyrene‐Phe‐Phe backbone has emerged as an intriguing self‐assembly motif, as our previous researches have demonstrated its great potential for intracellular assembly.^[^ ^50^, ^51^, ^52^, ^53^, ^54^ ^]^ Following this approach, we designed a supramolecular monomer containing the Pyrene‐Phe‐Phe peptide backbone. We conjugated acetazolamide ligand to the lysine residue to target CAIX on cancerous membranes as a lysosome trafficking enzyme, yielding **Py‐FFK‐Az** (Scheme 1a). To investigate the effect of self‐assembly propensity on LYTAC, we also designed a control group using the Pyrene‐Gly‐Gly backbone, which possesses weak self‐assembly propensity, yielding **Py‐GGK‐Az** (Scheme 1b). To generate Supra‐LYTAC, we designed additional peptides capable of interacting with POI. Since supramolecular co‐assembly can be utilized to fabricate unique nanostructure containing multiple functional groups on supramolecular surface, we designed **Py‐FFK‐POI**, expecting to co‐assemble with **Py‐FFK‐Az** (Lysosome trafficking monomer). To validate the feasibility of Supra‐LYTAC, we chose streptavidin as a model POI due to its well‐characterized high affinity with biotin.^[^ ^55^ ^]^ Accordingly, biotin was conjugated to the residual lysine, yielding **Py‐FFK‐Biotin** and **Py‐GGK‐Biotin**. All peptides were synthesized using solid‐phase peptide synthesis and characterized by MALDI‐TOF and analytical HPLC (Figure S1–S12, Supporting Information).

**Figure 1:**
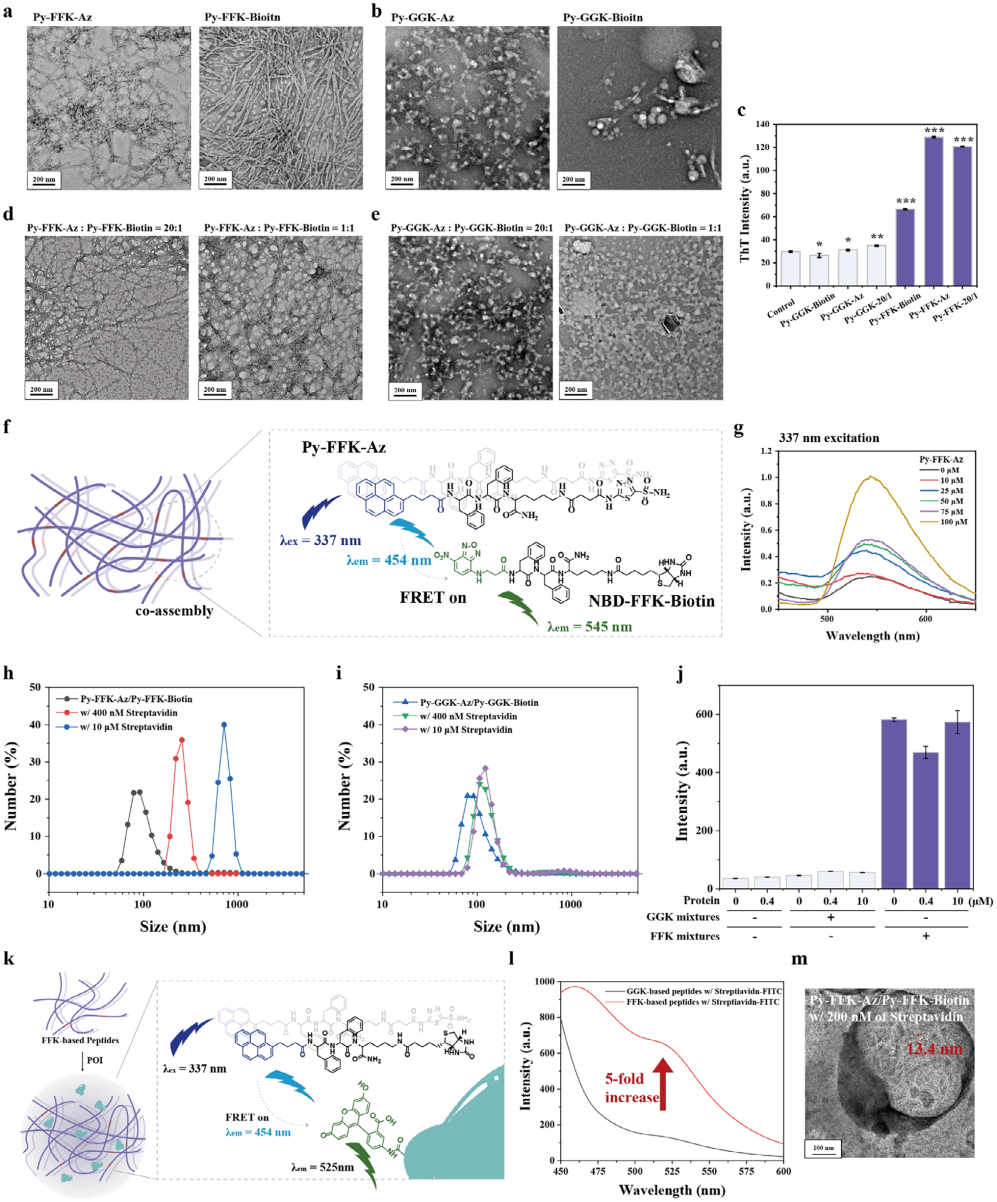
a,b) TEM image showing self‐assembly morphology for a) **Py‐FFK‐Az** and **Py‐FFK‐Biotin** and b) **Py‐GGK‐Az** and **Py‐GGK‐Biotin**; c) ThT assay for FFK‐and GGK‐based peptides to confirm amyloid fiber formation; d,e) TEM image showing co‐assembled nanostructure with different molar ratio for d) FFK‐ and e) GGK‐based peptides; f) Illustration showing FRET phenomenon between pyrene excimer and NBD moiety due to the co‐assembly; g) Fluorescence measurement for **NBD‐FFK‐Biotin** in **Py‐FFK‐Az** concentration dependent manner. h,i) DLS analysis for h) **Py‐FFK‐Az**/**Py‐FFK‐Biotin** and i) **Py‐GGK‐Az**/**Py‐GGK‐Biotin** (20/1 ratio) with streptavidin in concentration dependent manner; j) ThT analysis after streptavidin incubation toward GGK mixtures (5 µm **Py‐GGK‐Biotin** and 100 µm **Py‐GGK‐Az**) and FFK mixtures (5 µm **Py‐FFK‐Biotin** and 100 µm **Py‐FFK‐Az**). The fluorescence at 482 nm was plotted; k) Schematic illustration showing POI interaction with Supra‐LYTAC. FFK‐based peptides contains high capability to interact with POI, resulting in close proximity between the two probes; l) FRET signal observation at 360 nm excitation for streptavidin‐FITC with FFK‐ or GGK‐based peptides co‐incubation; m) TEM analysis for **Py‐FFK‐Az**/**Py‐FFK‐Biotin** (20/1 ratio) with 200 nm of streptavidin. Data are presented as mean ± SD (*n* = 3). ^*^ *P* < 0.05, ^**^ *P* < 0.01, and ^***^ *P* < 0.001 from student's *t*‐test.

Initially, we characterized the self‐assembly propensity of each synthesized peptide by measuring critical aggregation concentrations (CAC) using steady‐state fluorescence of pyrene.^[^ ^56^ ^]^ The CAC values were determined to be 43, 12, 9, and 7 µm for **Py‐FFK‐Az**, **Py‐FFK‐Biotin**, **Py‐GGK‐Az**, and **Py‐GGK‐Biotin**, respectively (Figure S13, Supporting Information). While no pyrene excimer peak for **Py‐FFK‐Az** was observed in DMSO solution indicative of the molecularly dissolved state, it appeared at 100 µm in aqueous solution, providing evidence of self‐assembly in aqueous conditions (Figure S14a,b, Supporting Information). In contrast to **Py‐FFK‐Az**, **Py‐GGK‐Az** showed no pyrene excimer peak above the CAC, indicating weak self‐assembly propensity (Figure S14c,d, Supporting Information). Transmission electron microscopy (TEM) was used to visualize the self‐assembly morphology. TEM images revealed that **Py‐FFK‐Az** and **Py‐FFK‐Biotin** assembled into nanofibers with diameters of ≈11 nm (**Figure** **1**a; Figure S15a, Supporting Information), while **Py‐GGK‐Az** formed worm‐like nanosphere structures, and **Py‐GGK‐Biotin** formed only nanospheres (Figure 1b). To further investigate the self‐assembly behavior of FFK‐ and GGK‐ based peptides, we exploited thioflavin T (ThT) assay, which is a common probe to monitor the amyloid fiber formation.^[^ ^57^ ^]^ A significant increase of ThT fluorescence was observed in the peptide solution composed of FFK‐based peptides (Figure 1c; Figure S16, Supporting Information). However, GGK‐based peptides showed no notable ThT fluorescence signal. Circular dichroism (CD) spectrum for FFK mixtures showed negative band ≈210–220 nm, and positive band ≈195–200 nm, implying *β*‐sheet structure (Figure S17a, Supporting Information).^[^ ^58^ ^]^ FT‐IR for FFK mixtures confirmed characteristic amide I band at 1633 cm^−1^, denoting the parallel *β*‐sheet conformation (Figure S17b, Supporting Information).^[^ ^59^, ^60^ ^]^ However, no characteristic peak was observed in GGK mixtures. These results suggest that FFK‐based peptides possess much higher self‐assembly propensity than GGK‐based peptides due to enhanced secondary interactions among peptide backbones, including hydrophobic interactions, π–π stacking, and hydrogen bonding.

Recent studies have highlighted the significance of homogeneous co‐assembled nanostructures comprising two functional peptides, as they offer a straightforward approach to optimize ligand tunability on supramolecular surfaces.^[^ ^25^, ^34^ ^]^ Following this approach, we developed a co‐assembled bifunctional nanostructure to construct Supra‐LYTAC. We first investigated the self‐assembly morphology of **Py‐FFK‐Az** combined with 5% and 50% **Py‐FFK‐Biotin**. The resulting homogeneous nanofiber structure demonstrated that co‐assembly between the two peptide backbones was the dominant process (Figure 1d; Figure S15b, Supporting Information). Similarly, when **Py‐GGK‐Az** was combined with 5% and 50% **Py‐GGK‐Biotin**, the spherical morphology remained unchanged (Figure 1e). Furthermore, the addition of 5% Py‐FFK‐Biotin and Py‐GGK‐Biotin did not alter ThT fluorescence compared to pure **Py‐FFK‐Az** and **Py‐GGK‐Az** (Figure 1c), indicating that POI‐interacting peptides did not affect the self‐assembly morphology.

To further explore the co‐assembly process, we synthesized **NBD‐FFK‐Biotin**, which is functionalized by 4‐chloro‐7‐nitrobenzofurazan (NBD) instead of pyrene at *N*‐terminal (Figure 1f). **NBD‐FFK‐Biotin** serves as a fluorescence resonance energy transfer (FRET) acceptor for pyrene excimer due to its spectral overlap (Figure S18, Supporting Information).^[^ ^61^ ^]^ We observed that **NBD‐FFK‐Biotin** fluorescence (λ_em_ = 545 nm) at 337 nm excitation increased proportionally with **Py‐FFK‐Az** concentration (Figure 1g), indicating close proximity between the two probes and confirming FRET phenomenon in the co‐assembled nanostructure. CLSM image showed that the acceptor fluorescence gradually increased with the high overlap between two probes when the **Py‐FFK‐Az** concentration increased (Figure S19, Supporting Information). All results suggest that our system could generate homogenous co‐assemble nanostructure composed of two different functional peptides, which are functionalized by CAIX targeting and POI interacting ligand.

To investigate the nanocomplex formation between our peptide assembly and POI, we first examined the interaction between streptavidin and two peptide systems: **Py‐FFK‐Az/Py‐FFK‐Biotin** and **Py‐GGK‐Az/Py‐FFK‐Biotin** (20:1 ratio) using dynamic light scattering (DLS). For FFK‐based peptides, increasing streptavidin concentration led to a significant increase in hydrodynamic size, reaching up to 1000 nm (Figure 1h; Figure S20, Supporting Information). In contrast, GGK‐based peptides showed negligible size changes, suggesting that the peptide backbone composition significantly influences the formation of supramolecular chimeric nanostructures with POI (Figure 1i; Figure S20, Supporting Information). These findings indicate that FFK‐based peptides can uniquely interact with POI to form nanocomplexes when nanofibrous self‐assembly occurs.

To further explore the mechanism of POI‐nanocomplex formation, we performed ThT assays on the supramolecular chimeric nanostructures. We observed a bathochromic shift in ThT fluorescence with increasing streptavidin concentration, indicating the formation of large nanocomplex structures (Figure 1j; Figure S21, Supporting Information). However, overall ThT fluorescence is comparable regardless of streptavidin, suggesting that the internal packing structure remained unaffected by POI. To validate the nanocomplex formation between self‐assembled structures and proteins, we conducted FRET experiments using streptavidin‐FITC. We hypothesized that pyrene excimer emission could transfer to streptavidin‐FITC, resulting in Fluorescein emission when self‐assembly structures and proteins co‐assembled (Figure 1k). FRET experiments at 337 nm excitation (corresponding to pyrene absorption) revealed that FFK‐based peptides exhibited significantly stronger FRET signals compared to GGK‐based peptides, indicating that self‐assembly propensity influences co‐assembly with POI (Figure 1l; Figure S22, Supporting Information). To further confirm that protein co‐assembly depends on the nanofibrous self‐assembly of FFK‐based peptides, we conducted concentration‐dependent FRET experiments with **Py‐FFK‐Az**. Weak FRET fluorescence was observed below the critical aggregation concentration (CAC), while a significant fluorescence increase occurred above CAC, confirming that self‐assembly is a prerequisite for POI co‐assembly (Figure S23, Supporting Information). Finally, we visualized the supramolecular chimeric structures formed with streptavidin by TEM. At 200 nm streptavidin concentration, FFK‐based peptides predominantly formed supramolecular nanocomplexes with diameters of 300 nm (Figure 1m). Inside the nanocomplexes, we observed nanofiber with a diameter of ≈13 nm, which is consistent with our observation in TEM at Figure 1a. However, il‐defined structures were investigated with 100 and 50 nm streptavidin, suggesting POI content is crucial to generate nanocomplex through supramolecular interaction (Figure S24, Supporting Information). In contrast to FFK‐based peptides, GGK‐based peptides displayed small aggregated particles similar to their morphology prior to protein incubation (Figure S25, Supporting Information). This pattern persisted across streptavidin concentrations up to 10 µm. Overall, these results demonstrate that FFK‐based peptides, through secondary interactions between peptide backbones, can effectively interact with POI to generate integrated nanocomplexes, a capability not observed in GGK‐based peptides.

### 2.2 Feasibility Study for Supra‐LYTAC Using Streptavidin‐FITC as a Model Protein

Based on the observed differences in supramolecular chimeric nanostructure formation, we next evaluated the feasibility of Supra‐LYTAC using streptavidin‐biotin chemistry. We selected HeLa and NIH/3T3 cell lines for this study, as our previous research demonstrated that CAIX expression was 3‐fold higher in HeLa cells compared to NIH/3T3 cells.^[^ ^46^ ^]^ Notably, we hypothesized that acetazolamide‐conjugated peptides would provide both lysosomal trafficking and tumor targeting capabilities through their interaction with cancerous CAIX. Initially, we verified the cytotoxicity of each designed peptides by MTT assay and confirmed that all synthesized peptides were non‐toxic (Figure S26, Supporting Information).

We then investigated POI uptake by incubating cells with our synthesized peptides and streptavidin‐FITC. When HeLa cells were treated with **Py‐FFK‐Biotin**, significant POI internalization occurred above the CAC (Figure S27a, Supporting Information), while no streptavidin‐FITC uptake was observed below the critical aggregation concentration of **Py‐FFK‐Biotin** (CAC, ≈12 µm). To isolate the effects of Supra‐LYTAC from **Py‐FFK‐Biotin** self‐assembly, we set the **Py‐FFK‐Biotin** concentration at 5 µm (below CAC) for subsequent experiments. In contrast, **Py‐FFK‐Az** did not affect POI internalization regardless of the CAC due to the absence of POI interacting ligand (Figure S27b, Supporting Information). Similarly, neither **Py‐GGK‐Az** nor **Py‐GGK‐Biotin** facilitated streptavidin‐FITC uptake, even at concentrations above their CAC (Figure S27c,d, Supporting Information). These results demonstrate that both membrane‐proximal self‐assembly formation and supramolecular interactions between peptide backbones are crucial factors for successful Supra‐LYTAC implementation.

**Figure 2:**
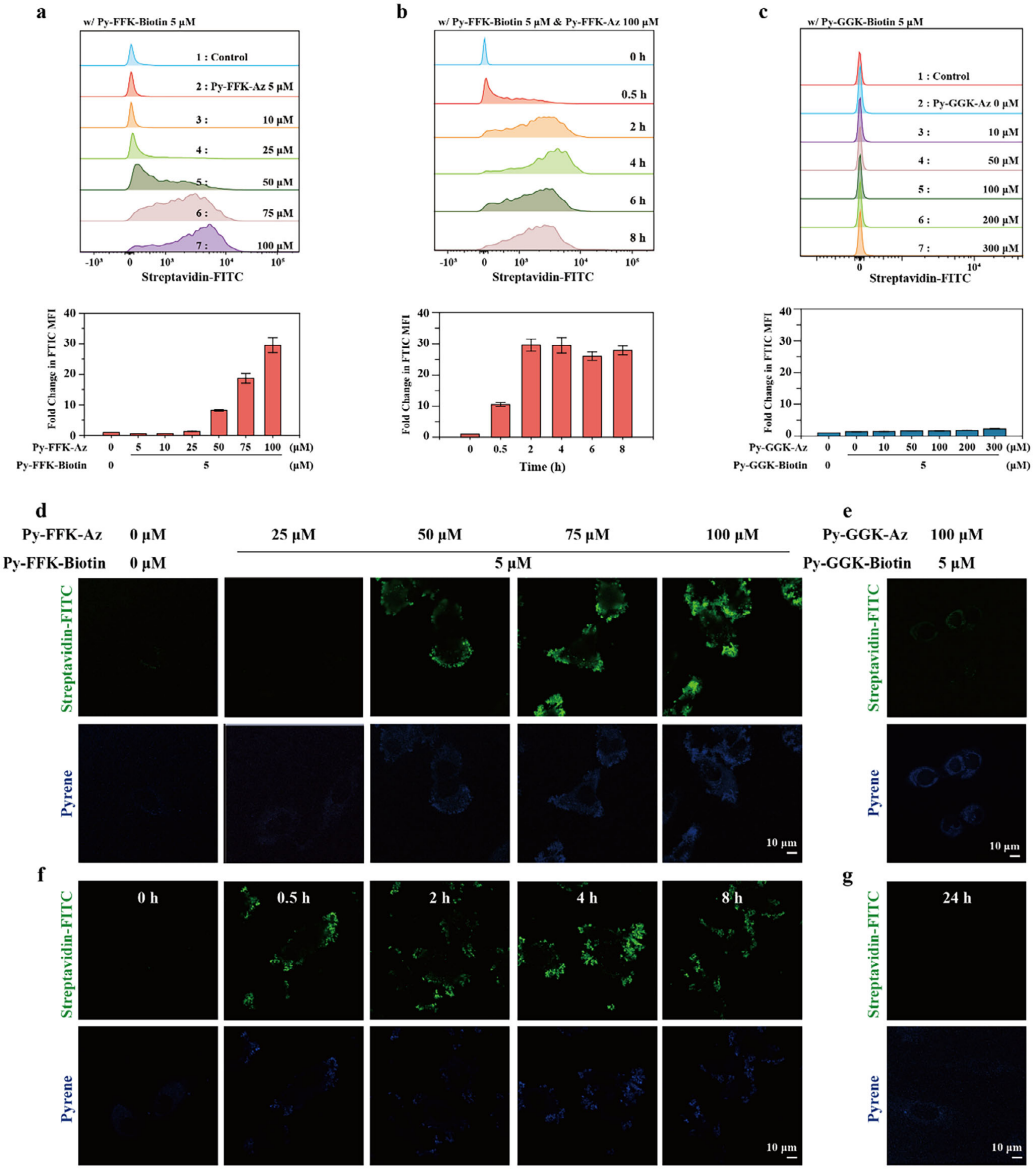
a) Flow cytometry analysis for streptiavidn‐FITC uptake into HeLa. The solution for 400 nm streptavidin‐FITC, 5 µm **Py‐FFK‐Biotin,** and **Py‐FFK‐Az** at varying concentration were prepared. The solution was incubated toward HeLa for 4‐h. After wash, the collected cells were measured by flow cytomertry. Fold change of streptaivdin‐FITC uptake were analyzed by FlowJo software; b) The solution for 400 nm streptavidin‐FITC, 5 µm **Py‐FFK‐Biotin,** 100 µm **Py‐FFK‐Biotin** was incubated toward HeLa in time dependent manner. After wash, the collected cells were measured by flow cytomertry. Fold change of streptaivdin‐FITC uptake were analyzied by FlowJo software. It was saturated from 2 h; c) Fold change of streptaivdin‐FITC with 5 µm **Py‐GGK‐Biotin** in **Py‐GGK‐Az** concentration dependent manner after 4 h incubation. The procedure is same with FFK mixtures; d‐e) CLSM image showing streptavidin‐FITC internalization in d) concentration dependent manner for FFK‐based peptide and e) GGK‐based peptide after 4 h incubation; f,g) CLSM image showing streptavidin‐FITC internalization in f) time dependent manner for FFK‐based peptides (5 µm **Py‐FFK‐Bioitn** and 100 µm **Py‐FFK‐Az**) and g) GGK‐based peptides after 24 h incubation (5 µm **Py‐GGK‐Biotin** and 100 µm **Py‐GGK‐Az**). Data are presented as mean ± SD (*n* = 3).

We investigated streptavidin‐FITC uptake in HeLa cells using a mixture for **Py‐FFK‐Az** and **Py‐FFK‐Biotin** to confirm in situ Supra‐LYTAC activity. Given that membrane‐proximal self‐assembly is crucial for Supra‐LYTAC function, we expected that **Py‐FFK‐Az** would facilitate supramolecular chimeric nanostructure formation through the interaction with membrane protein, CAIX. When we measured streptavidin‐FITC uptake at varying **Py‐FFK‐Az** concentrations while maintaining **Py‐FFK‐Biotin** at 5 µm to exclude pre‐assembly‐mediated POI uptake, it is revealed significant POI internalization in HeLa cells beginning at 50 µM **Py‐FFK‐Az** (above the CAC, ≈43 µm) from the flow cytometry analysis (**Figure** **2**a). Confocal laser scanning microscopy (CLSM) confirmed the flow cytometry results, showing gradual increases in POI fluorescence starting at 50 µm **Py‐FFK‐Az** (Figure 2d). Additionally, we measured streptavidin‐FITC uptake in **Py‐FFK‐Biotin** concentration dependent manner with 100 µm **Py‐FFK‐Az**. We observed that ≈75‐fold higher MFI intensity compared to untreated group (Figure S28, Supporting Information). Notably, it was 3‐fold higher intensity compared to 100 µm Py‐FFK‐Biotin single treatment (Figure S27a, Supporting Information), showing the importance for CAIX‐Targeting via **Py‐FFK‐Az** co‐treatment. Time‐dependent analysis using flow cytometry revealed POI internalization beginning at 30 min, reaching maximum levels at 2 h, and persisting beyond 8 h (Figure 2b). CLSM imaging similarly showed notable streptavidin‐FITC signals inside HeLa cells from 30 min onward (Figure 2f). In addition, we observed that streptavidin‐FITC fluorescence signal was significantly diminished from 36 h. From 48 h incubation, pyrene fluorescence from Supra‐LYTAC was also reduced, implying our peptide‐based system with POI could be degraded in longer incubation (Figure S29, Supporting Information). To evaluate cancer targeting capability conferred by acetazolamide‐conjugated peptides, we compared POI internalization between HeLa cells and NIH/3T3 cells, which express lower levels of CAIX. The significantly reduced POI uptake in NIH/3T3 cells suggests the potential for spatiotemporal control of Supra‐LYTAC (Figure S30, Supporting Information). In contrast to FFK‐based peptides, GGK‐based peptides showed no POI uptake capability. When cells were treated with 5 µm **Py‐GGK‐Biotin** and increasing concentrations **of Py‐GGK‐Az** (up to 300 µm), no significant POI uptake was observed (Figure 2c), as confirmed by CLSM imaging (Figure 2e; Figure S31, Supporting Information). To rule out potential concentration‐dependent effects, we also tested POI uptake with 100 µm **Py‐GGK‐Biotin** and varying concentrations of **Py‐GGK‐Az**. Despite being above the CAC for **Py‐GGK‐Biotin**, this combination still showed negligible POI uptake (Figure S32a, Supporting Information). Time‐course flow cytometry analysis of GGK‐based peptides similarly showed no significant fluorescence increase over 24 h (Figure 2g; Figure S32b, Supporting Information). Similarly, the combination with **Py‐FFK‐Az** and biotin in the absence of peptide backbone showed no significant POI uptake (Figure S33, Supporting Information). These results highlight the crucial role of protein‐peptide self‐assembly in Supra‐LYTAC function, particularly emphasizing the importance of supramolecular nanofibrous structures with specific secondary interactions for effective POI capture.

**Figure 3:**
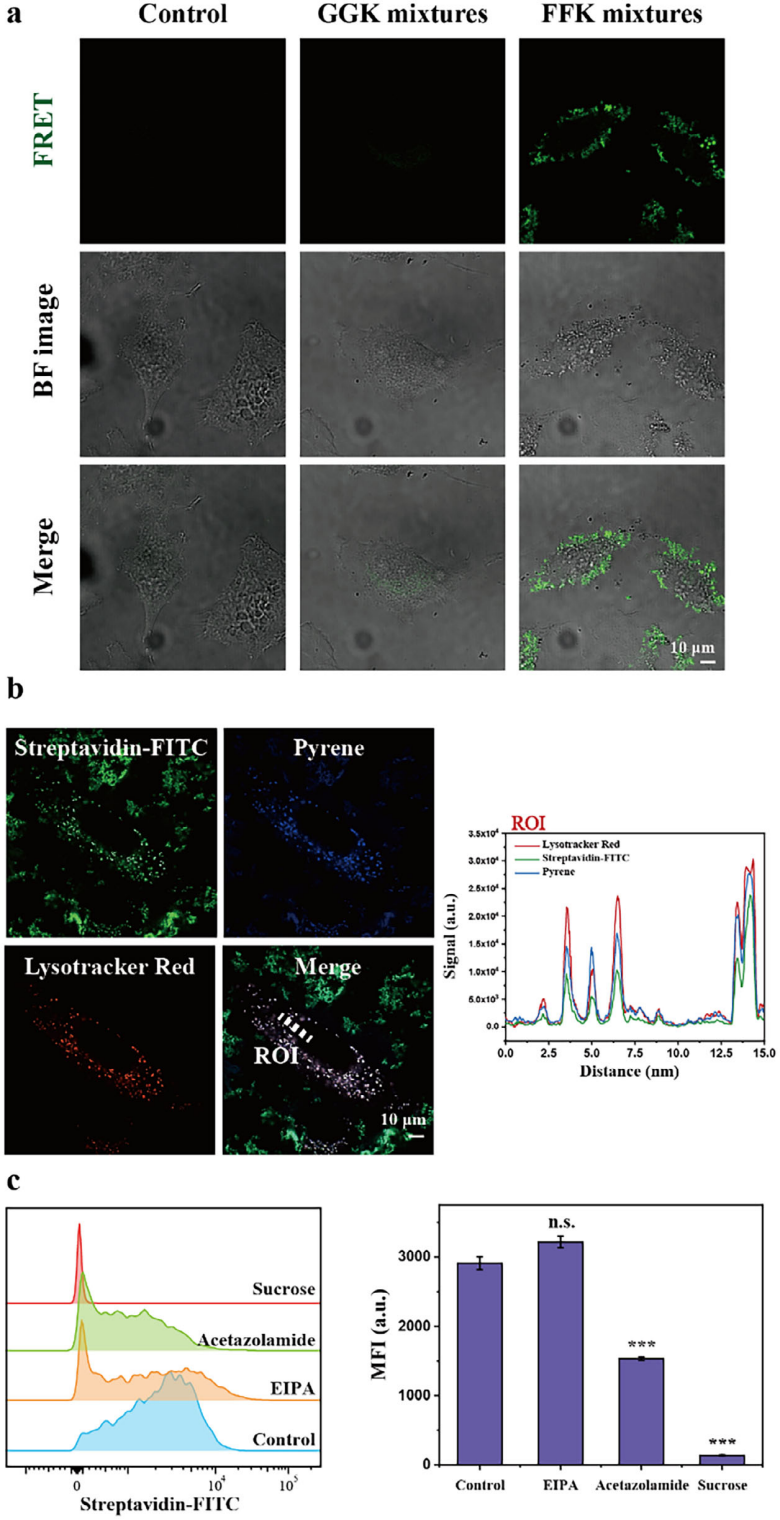
a) FRET observation after incubating POI with peptides mixture after 4 h incubation (Control: 400 nm streptavidin‐FITC, GGK mixtures: 400 nm streptavidin‐FITC with 5 µm **Py‐GGK‐Biotin** and 100 µm **Py‐GGK‐Az**, FFK mixtures: 400 nm streptavidin‐FITC with 5 µm **Py‐FFK‐Biotin** and 100 µm **Py‐FFK‐Az**); b) CLSM image showing co‐localization for Streptavidin‐FITC with Lysotracer Red and pyrene after 16 h. High overap was observed in ROI profile; c) MFI analysis of internalized Streptaividn‐FITC with the co‐treatment of endocytosis inhibitor. Data are presented as mean ± SD (*n* = 3). ^*^ *P *< 0.05, ^**^ *P* < 0.01, and ^***^ *P* < 0.001 from student's *t*‐test.

We anticipated that nanofibers formed through interaction with CAIX on cancer cell membranes would similarly engage with POI to construct nanocomplexes. To validate the nanocomplex formation between self‐assembled structures and proteins near cancerous membrane, we additionally obtained CLSM image after incubating streptavidin‐FITC and the peptides mixture toward HeLa to visualize FRET phenomenon. After 2 h incubation, we observed significant FRET signal was observed in the FFK‐based peptides solution, while GGK based peptide showed neglective FRET signal (**Figure** **3**a). This result indicates that co‐assembly with POI is more advantage for FFK‐based peptides compared to GGK‐based peptides due to increased self‐assembly propensity of nanofibrous structure constructed near cancerous membrane.

We next examined the cellular localization of Supra‐LYTAC using CLSM. After 16 h, ROI profile analysis revealed significant co‐localization of Streptavidin‐FITC, Pyrene, and Lysotracker Red, confirming lysosomal targeting of Supra‐LYTAC (Figure 3b). To understand the lysosomal trafficking mechanism, we conducted inhibitor studies. POI uptake was significantly reduced by acetazolamide (a CAIX inhibitor), while EIPA (a micropinocytosis inhibitor) showed no effect compared to the negative control (Figure 3c). Notably, sucrose treatment (a clathrin‐mediated endocytosis inhibitor) substantially reduced POI uptake, consistent with previous reports that CAIX undergoes clathrin‐mediated endocytosis.^[^ ^62^, ^63^ ^]^ These findings demonstrate that Py‐FFK‐Az in Supra‐LYTAC interacts with CAIX on cancer cell membranes to facilitate POI internalization.

### 2.3 Membrane Anchoring Protein Degradation Study for Supra‐LYTAC

**Figure 4:**
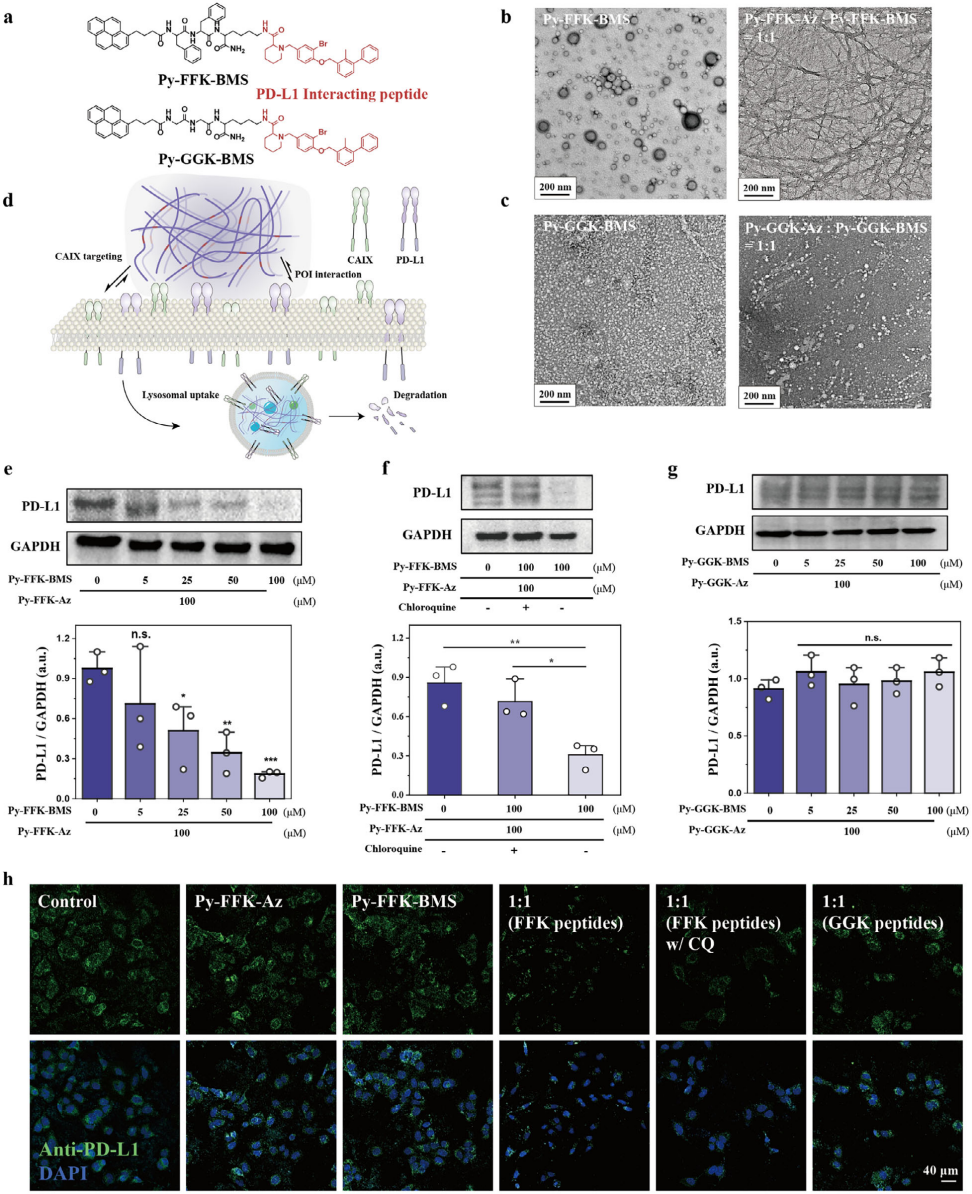
a) Monomer design for PD‐L1 interacting peptide; b) TEM analysis for **Py‐FFK‐BMS**, and co‐assembly with **Py‐FFK‐Az** in ratio of 1:1; c) TEM analysis for **Py‐GGK‐BMS**, and co‐assembly with **Py‐GGK‐Az** in ratio of 1:1 showing nanosphere structure; d) Illustration showing Supra‐LYTAC for membrane‐anchoring protein internalization into lysosome; e) WB analysis for PD‐L1 degradation for **Py‐FFK‐BMS** concentration dependent manner with 100 µm **Py‐FFK‐Az** after 24 h incubation; f) WB analysis for PD‐L1 degradation after incubating **Py‐FFK‐BMS** and **Py‐FFK‐Az** with or without chloroquine (lysosomal inhibitor) after 24 h incubation g) WB analysis for PD‐L1 degradation after incubating 100 µm **Py‐GGK‐Az** in **Py‐GGK‐BMS** concentration dependent manner after 24 h incubation; h) IF image showing PD‐L1 degradation toward HeLa after 24 h incubation with 100 µm concentration of each peptides. Data are presented as mean ± SD. ^*^ *P *< 0.05 and ^**^ *P* < 0.01 from student's *t*‐test.

To validate the feasibility of membrane anchoring protein degradation, we selected PD‐L1, a representative immune checkpoint inhibitor, as our target protein. We synthesized **Py‐FFK‐BMS** and **Py‐GGK‐BMS** by conjugating **BMS‐8**, a known PD‐L1 binding ligand, to the *C*‐terminal of our self‐assembling peptides instead of biotin with the core scaffold for binding remaining intact^[^ ^64^, ^65^, ^66^ ^]^ (**Figure** **4**a). Initially, the CAC value was measured using pyrene emission method. It was determined to be 48 and 30 µm for **Py‐FFK‐BMS** and **Py‐GGK‐BMS**, respectively (Figure S34, Supporting Information). In DMSO solution, both **Py‐FFK‐BMS** and **Py‐GGK‐BMS** did not represent excimer peak indicative of molecularly dissolvent state (Figure S35, Supporting Information). However, a strong excimer peak at 462 nm was observed only for **Py‐FFK‐BMS** in aqueous solution (Figure S35b, Supporting Information). TEM imaging was used to visualize the self‐assembly morphology. When mixed at a 1:1 ratio, FFK‐based peptides formed homogeneous nanofiber structures, despite **Py‐FFK‐BMS** alone forming nanospheres (Figure 4b). In contrast, GGK‐based peptides maintained their nanosphere morphology in the 1:1 mixture, with **Py‐GGK‐BMS** showing small nanosphere aggregates (Figure 4c). Similar to above biotin‐avidin chemistry, we hypothesized that Supra‐LYTAC composed of **Py‐FFK‐Az** and **Py‐FFK‐BMS** would demonstrate superior performance compared to the combination of **Py‐GGK‐Az** and **Py‐GGK‐BMS** due to differences in self‐assembly propensity (Figure 4d).

We next evaluated PD‐L1 degradation using Western blot (WB) analysis. Initial WB characterization revealed that HeLa cells expressed 10‐fold higher PD‐L1 levels compared to NIH/3T3 cells (Figure S36, Supporting Information). Initially, we verified BMS‐conjugated peptides were non‐toxic by MTT assay (Figure S37, Supporting Information). We then assessed PD‐L1 degradation in HeLa cells after 24‐h treatment with 100 µm **Py‐FFK‐Az** and varying concentrations of **Py‐FFK‐BMS**. The results showed that PD‐L1 degradation efficiency increased proportionally with **Py‐FFK‐BMS** concentration (Figure 4e). Especially, significant PD‐L1 degradation was observed even below the CAC for **Py‐FFK‐BMS** (≈48 µm, Figure S34, Supporting Information), indicating the effect for CAIX‐Targeting toward PD‐L1 degradation for Supra‐LYTAC. To further assess the effect for **Py‐FFK‐Az**, we conducted WB analysis with 100 µm **Py‐FFK‐BMS** at varying concentration for **Py‐FFK‐Az**. WB results suggest PD‐L1 degradation was highly mitigated in **Py‐FFK‐BMS** single treatment group (Figure S38, Supporting Information). However, PD‐L1 degradation level increased when **Py‐FFK‐Az** concentration increases. PD‐L1 degradation was inhibited by co‐treatment with CAIX inhibitor, confirming the role of CAIX‐mediated endocytosis in lysosomal uptake (Figure S39, Supporting Information). To further validate lysosome‐dependent protein degradation, we conducted WB analysis with chloroquine, a lysosomal inhibitor. Co‐treatment with chloroquine significantly reduced PD‐L1 degradation at the 1:1 ratio of **Py‐FFK‐Az** and **Py‐FFK‐BMS** (Figure 4f). In contrast to FFK‐based peptides, GGK‐based peptides showed no significant PD‐L1 degradation when cells were treated with 100 µm **Py‐GGK‐Az** and increasing concentrations of **Py‐GGK‐BMS** up to a 1:1 ratio (Figure 4g). These findings were corroborated by immunofluorescence (IF) imaging of PD‐L1, which showed significantly reduced fluorescence signals with 1:1 **Py**‐**FFK‐Az:Py‐FFK‐BMS** treatment (Figure 4h). Single treatments with **Py‐FFK‐Az** or **Py‐FFK‐BMS**, and 1:1 GGK‐based peptide combinations showed fluorescence comparable to control groups. Furthermore, PD‐L1 fluorescence was restored when chloroquine was co‐administered with the 1:1 **Py‐FFK‐Az:Py‐FFK‐BMS** mixture, confirming lysosomal involvement in PD‐L1 degradation. These results collectively demonstrate that Supra‐LYTAC systems incorporating strong secondary interactions between peptide backbones can effectively achieve membrane‐anchored protein degradation.

**Figure 5:**
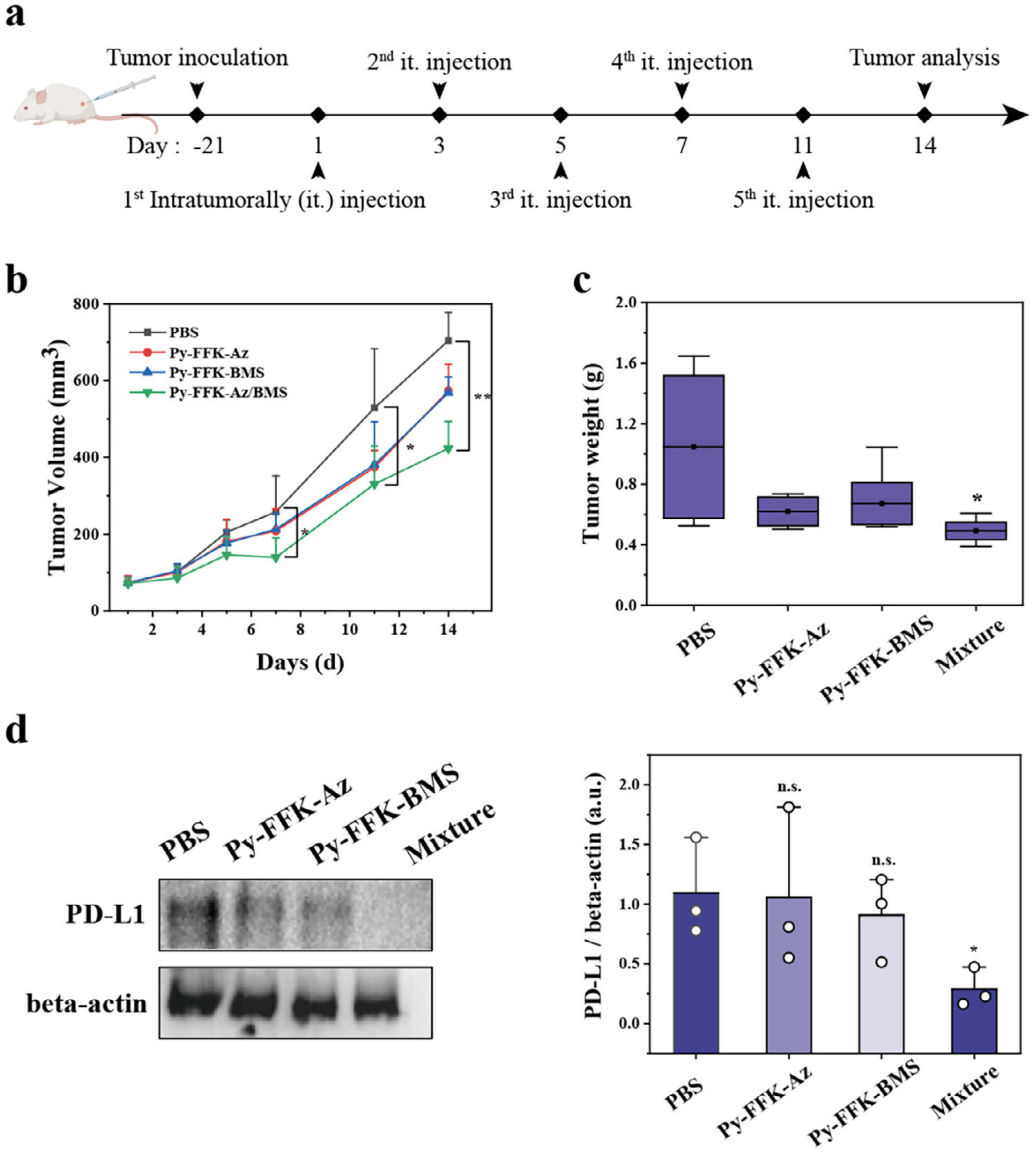
a) Schematic illustration showing the time course of Supra‐LYTAC treatment in xenograft model. Each group of mice were injected periodically by intratumoral administration; b) Tumor volume measurement of periodic treatment for PBS, **Py‐FFK‐Az**, **Py‐FFK‐BMS**, and **Py‐FFK‐Az/Py‐FFK‐BMS**; c) Average tumor weight measurement of different group; d) WB image showing PD‐L1 degradation after periodic treatment for different group and its quantitative graph for PD‐L1 expression level of WB image. Data are presented as mean ± SD. ^*^ *P *< 0.05 and ^**^ *P* < 0.01 from one‐way ANOVA with Tukey's post‐hoc test.

We next evaluated the efficacy of Supra‐LYTAC in vivo using a 4T1 xenograft model. First, we verified the feasibility toward 4T1, resulting in significant POI uptake and PD‐L1 degradation comparable to that of HeLa (Figure S40, Supporting Information). We established 4T1 bearing xenograft model by a subcutaneously injection into the mice flank. Based on our hypothesis that PD‐L1 degradation by Supra‐LYTAC could enhance immunogenic response, we investigated its anti‐tumor efficacy. Since we confirmed that Supra‐LYTAC could be spatiotemporally activated when tumor targetable peptide (**Py‐FFK‐Az**) and POI interacting peptide (**Py‐FFK‐BMS**) are co‐treated in vitro, we set the four groups (PBS, **Py‐FFK‐Az**, **Py‐FFK‐BMS**, and 1:1 ratio of **Py‐FFK‐Az/Py‐FFK‐BMS**) to confirm the importance of co‐treatment of both peptides. Each group are periodically treated with the concentration of 2.5 mg kg^−1^ into the mouse intratumorally when tumor volume reached to 80 mm^3^ (**Figure** **5**a). During the treatment, we measured a tumor volume of different group, demonstrating that simultaneous treatment for **Py‐FFK‐Az** and **Py‐FFK‐BMS** showed highest efficacy to reduce tumor volume compared to other groups (Figure 5b). After reaching the tumor size ≈800 mm^3^, we sacrificed the mouse and analyzed tumor weight. In consistent with the results of tumor size, it was revealed that tumor size was significantly reduced when **Py‐FFK‐Az** and **Py‐FFK‐BMS** are co‐treated (Figure 5c; Figure S41, Supporting Information). Otherwise, the body weight of every mouse was not significantly changed after the periodic treatment, showing biocompatibility of Supra‐LYTAC (Figure S42, Supporting Information). To reveal anti‐tumor efficacy in detail, we conducted WB analysis for checking PD‐L1 expression level in each group. We observed that PD‐L1 levels are highly decreased in co‐treatment group for **Py‐FFK‐Az** and **Py‐FFK‐BMS** compared to each group while significant PD‐L1 degradation was not observed compared to PBS in the single treatment (Figure 5d). Overall results indicated that Supra‐LYTAC could be exerted in animal model to show significant anti‐tumor efficacy by degrading PD‐L1 in the tumor tissue.

## 3 Conclusion

In conclusion, this study represents development of cancer specific CAIX targeting Supra‐LYTAC for TPD. Considering the unique feature for supramolecular self‐assembly such as a dynamic properties and multivalent binding characteristics, we designed and synthesized two hetero‐functionalized self‐assemble monomers containing CAIX‐targeting and POI‐interacting ligand. Two monomer co‐assembled each other to yield nanofibrous supramolecular chimeric nanostructure (Supra‐LYTAC) in aqueous solution. In addition, we observed that our peptide assembly could interact with POI to occur the nanocomplex formation. To get mechanistic insight for Supra‐LYTAC, we further conducted comparative analysis of FFK‐based peptides and GGK‐based peptides, which differ in self‐assembly propensity. It suggests that secondary interaction between peptide backbones plays a pivotal role in the establishment for integrated nanocomplex through the efficient POI capture. In cellular environment, we confirmed the membrane‐proximal self‐assembly construction by CAIX‐targeting and following the nanocomplex formation through POI capture near the cellular membrane. As a result of generation of Supra‐LYTAC near cancerous membrane, POI trafficking into lysosomes was significantly implemented. However, no POI uptake was observed in the peptide mixture possessing weak self‐assembly propensity, implying the significance of specific secondary interaction. Additionally, we validated the feasibility for Supra‐LYTAC toward the membrane anchoring protein, showing efficient PD‐L1 degradation from the peptide self‐assembly containing a strong secondary interaction. Overall, our findings confirmed the feasibility of Supra‐LYTAC toward extracellular protein (Streptavidin) and membrane protein (PD‐L1). Finally, we revealed the therapeutical potential for Supra‐LYTAC by degrading PD‐L1 in xenograft model. This is the first demonstration of CAIX‐Targeting supramolecular nanofibrous lysosome‐targeting chimeras for targeted protein degradation, significantly expanding the category of LYTAC beyond conventional small molecule or antibody‐based chimeric structure. This study would provide various opportunities for the rational design of advanced supramolecular LYTAC in the future.

## Conflict of Interest

The authors declare no conflict of interest.

## Supporting information



Supporting Information

## Data Availability

The data that support the findings of this study are available in the supplementary material of this article.
