# Supplementary material for: Cancer Specific CAIX‐Targeting Supramolecular Lysosome‐Targeting Chimeras (Supra‐LYTAC) for Targeted Protein Degradation
Source: Adv Sci (Weinh). 2025 Apr 3;12(26):2503134. doi: 10.1002/advs.202503134 (PMC12245127; doi:10.1002/advs.202503134)
Supplement: Supplementary file 1 — Supporting Information [file ADVS-12-2503134-s001.pdf]

ADVANCED SCIENCE
Open Access

Supporting Information

for *Adv. Sci.*, DOI 10.1002/advs.202503134

Cancer Specific CAIX-Targeting Supramolecular Lysosome-Targeting Chimeras (Supra-LYTAC) for Targeted Protein Degradation

*Dohyun Kim, Gyeongseok Yang, Chaelyeong Lim, Gaeun Park, Jaemo Lee, Youjung Sim and Ja-Hyoung Ryu**

Cancer Specific CAIX-Targeting Supramolecular Lysosome-Targeting Chimeras (Supra-LYTAC) for Targeted Protein Degradation

Dohyun Kim, Gyeongseok Yang‡, Chaelyeong Lim, Gaeun Park, Jaemo Lee, Youjung Sim, and Ja-Hyoung Ryu*

Ulsan National Institute of Science and Technology (UNIST), Ulsan 44919, Republic of Korea

* Corresponding Author. Email: jhryu@unist.ac.kr

Table of Contents

1. **Experimental Sections**
2. **Supplementary Figures**
3. **Reference**

S1. Experimental Sections

S.1.1. Materials. Fmoc-protected amino acid and O-benzotrazole-N,N,N',N'-tetramethyluronium-hexafluorophosphatehexafluorophosphate (HBTU) were purchased from Apexbio. Rink amide MBHA resin, pyrene, and 5-(N-Ethyl-N-Isopropyl)amiloride (EIPA) were purchased from Sigma Aldrich. N, N-diisopropylethylamine (DIPEA), trifluoroacetic acid (TFA), sucrose, acetazolamide were purchased from TCI. Thioflavin T was purchased from Acros Organics. All the solvents were purchased from SAMCHUN chemicals. Streptavidin (pro-791-b) was purchased from Prospec. Streptavidin-FITC (7105-02) was purchased from Southern Biotech Anti-PD-L1 (ab205921), and Alexa Fluor-488 conjugated Goat Anti-Rabbit (ab150077) were purchased from Abcam. Anti-GAPDH (sc-32233) was purchased from Santa Cruz Biotechnology. Goat Anti-Rabbit IgG (H+L)-HRP Conjugate (1706515) and Goat Anti-Mouse IgG (H+L)-HRP conjugate (1721011) were purchased from Bio-Rad. 3-(4,5-dimethylthiazol-2-yl)-2,5-diphenyltetrazolium bromide (MTT) was purchased from ACROS Organics. LysoTracker^{TM} Red DND-99 was purchased from Thermo Fisher Scientific. For cell culture, Dulbecco's Modified Eagle's Medium (DMEM), Rosewell Park Memorial Institute (RPMI), fetal bovine serum (FBS), and penicillin/streptomycin were purchased from Gibco by Life technologies.

S.1.2. Synthesis for 5-amino-1,3,4-thiadiazole-2-sulfonamide (1). It was prepared by following previous literature[1]. Briefly, hydrochloric acid (2N, 50 ml) was added to a solution of N-(6-sulfamoyl-1,3,4-thiadiazol-2-yl) acetamide (5.0 g, 22.5 mmol) in 75 ml methanol. After that, the solution was refluxed for 18 h and then solvent was evaporated. The residue was purified with column chromatography on a silica gel. The product was obtained as a white solid. Yield: 3.27 g (80%). ^{1}H NMR (400 MHz, DMSO-d6): δ 8.05 (s, 2H), δ 7.80 (s, 2H)

S.1.3. Synthesis for 4-oxo-4-((5-sulfamoyl-1,3,4-thiadiazol-2-yl)amino)butanoic acid (2). It was prepared by following previous literature[1]. Briefly, succinic anhydride (0.22 g, 2.22 mmol) was added to a solution of Az-NH2 (0.40 g, 2.22 mmol) in *N,N*-dimethylformamide. After that, the solution was heated at 100˚C for 12 h. Then, the solvent was evaporated and the product was utilized in next step without further purification. Yield: 0.60 g (96%). ^{1}H NMR (400 MHz, DMSO-d6): δ 8.29 (s, 2H), δ 2.72 (t, 2H), δ 2.56 (t, 2H)

S.1.4. Synthesis for 3-((7-nitrobenzo[c][1,2,5] oxadiazol-4-yl)amino)propanoic acid (3). 6.25 g of β-alanine (3.25 g, 7 equiv.) and sodium bicarbonate (5.892 g, 7 equiv.) was dissolved in 30 mL of DI water. To a solution of 2 g of 4-Chloro-7-nitrobenzofurazan (10.02 mmol, 1 equiv.), it was added dropwise. After that, it was stirred at 70 °C for 1 h, and further stirred at 50 °C for another 5 h. After removing the solvent, cold water was added into the solution. Then, 36% HCl was slowly added until precipitate forms. After another 30 min, the precipitate was collected and used for the next reaction without any further purification. Yield: 1.622 g (64%). ^{1}H NMR (400 MHz, DMSO-d6): δ 12.40 (s, 1H), δ 9.44 (m, 1H), δ 8.50 (m, 1H), δ 6.42 (d, 1H), δ 3.63 (t, 2H), δ 2.68 (t, 2H)

S.1.5. Synthesis for 3-bromo-4-((2-methyl-[1,1'-biphenyl]-3-yl)methoxy)benzaldehyde (4). 710 mg of 3-bromo-4-hydroxybenzaldehyde (3.51 mmol, 1 equiv.), 696 mg of 3-hydroxymethyl-e-methylbiphenyl (3.51 mmol, 1 equiv.), and 1.02 g of triphenylphosphine (3.89 mmol, 1.1 equiv.) was dissolved in anhydrous THF. Then, 0.74 mL of diisopropyl azodicarboxylate (3.89 mmol, 1.1 equiv.) was added dropwise at 0°C . The reaction continued

at RT for 24 h. After the reaction, purification by flash chromatography (0-60%, ethyl acetate in hexane) was achieved to obtain the product. Yield: 416 mg (31.1%) ^{1}H NMR (400 MHz, DMSO-d6): δ 9.87 (s, 1H), δ 8.14 (d, 1H), δ 7.97 (dd,1H), δ 7.55−7.52 (m, 2H), δ 7.48−7.45 (m, 2H), δ 7.40−7.37 (m, 1H), δ 7.33−7.30 (m, 3H), δ 7.22 (dd, 1H), δ 5.39 (s, 2H), δ 2.23 (s, 3H)

S.1.6. Synthesis for 1-[[3-bromo-4-[(2-methyl[1,1'-biphenyl]-3-yl)methoxy]phenyl]methyl]-2-piperidinecarboxylic acid (5). 148 mg of **4** (0.39 mmol), 151 mg of pipecolic acid (1.17 mmol), 74 mg of sodium cyanoborohydride (1.17 mmol), and 2 drops of acetic acid were mixed in 4 mL DMF. After 3 h at 80℃, it was purified by flash chromatography to obtain the product. Yield: 80 mg (41.6%) ^{1}H NMR (400 MHz, DMSO-d6) 7.59 (d, 1H), 7.53 (dd, 1H), 7.49—7.24 (m, 8H), 7.21 (dd, 1H), 5.23 (s, 2H), 3.81 (d, 2H), 3.44 (d, 2H), 2.22 (s, 3H), 1.85—1.27 (m, 7H).

S.1.7. Peptide synthesis. All the peptides were synthesized using solid phase peptide synthesis. Briefly, MBHA rink amide resin (200 mg, 0.106 mmol) was swelled in DMF for 30 min, then Fmoc-deprotection was achieved using 20% piperidine in DMF for 40 min. After Fmoc deprotection, the following amino acid was loaded onto resin (0.53 mmol of amino acid, 0.53 mmol of HBTU, and 1.06 mmol of DIPEA) for 1 h. This step was repeated until obtaining desired sequence. After that, N-terminal capping moiety (1-Pyrenebutyric acid, 5 equiv.) was coupled to yield pyrene-conjugated peptides. For NBD-conjugated peptides, **3** was conjugated as N-terminal capping moiety. Then, the resin was cleaved by cocktail solution (TFA/Triisopropyl silane/H_2O = 95/2.5/2.5). After precipitation in cold ether, crude peptides were purified by High Performance Liquid Chromatography (HPLC, Agilent 1100 HPLC system and 1220 HPLC system with Eclipse SDB-C18 column). After that, 5 equiv. of biotin, **2**, or **5** was further conjugated to yield acetazolamide-, biotin-, or BMS-conjugated peptides. The peptides were purified by HPLC, and the mass of peptides was analyzed by Matrix-Assisted Laser Desorption/Ionization Time-of-Flight (MALDI-TOF/TOF, Ultraflex III, Bruker).

S.1.8. Co-assembly between peptide monomers. Each synthesized monomers are dissolved in DMSO to yield 10 mM stock solution. Stock solutions are mixed with different ratio for the following experiment conditions, and it was further diluted to yield working solutions.

S.1.9. UV/Vis measurement. To verify the self-assembly behavior, peptide stock solution (10 mM in DMSO) was diluted into DMSO and water to yield 100 μM working solution. After that, UV/Vis spectra was obtained using UV/Vis spectroscopy (V-670, JASCO)

S.1.10. Thioflavin T (ThT) assay. After self-assembly for each peptide, thioflavin T (1 mM stock solution in water) was diluted to peptide solution to make 50 μM working solution. Then, the solution was stirred at RT for 30 min to fully encapsulate ThT into self-assembly structure. The solution was monitored using fluorometer (F-7000, Hitachi) with the excitation of 450 nm

S.1.11. Critical aggregation concentrations (CAC) measurements. CAC for each synthesized peptide was measured by pyrene emission method[1]. Initially, 2 mM pyrene stock solution was prepared in ethanol. Then, it was further diluted into the peptide solution to yield 2 μM pyrene working solution. To fully encapsulate the pyrene into peptide assembly, it was further stirred for 30 min in RT. After that, the fluorescence for pyrene was recorded using

fluorometer (F-7000, Hitachi). The ratio for I_1 band and I_3 band was plotted to obtain CAC value of each peptide.

S.1.12. Transmission electron microscopy study. To confirm the peptide self-assembly nanostructures, 50 μM of peptide solution in water was prepared. Then, 8 μL was dropped onto a copper grid. After the droplet is fully dried, it was repeated once more. After that, negative staining was achieved using 2 wt% uranyl acetate solution for 10 min in RT. After washing using DI water, the self-assembly morphology was visualized using JEM-1400 by JEOL and morganini 268.

To visualize peptide-protein nanocomplex, streptavidin stock solution was added into the solution consisting of 50 μM peptide solution (mixture of Py-FFK-Az and Py-FFK-Biotin in 20:1 ratio) to obtain 400 nM and 10 μM solutions. Then, TEM sample was prepared with the same protocol of peptide self-assembly visualization.

S.1.13. Circular dichroism spectroscopy. 100 μM peptide solution was initially prepared. The solution was transferred into quartz cell with 1 mm path length (Hellma), and CD spectrum was recorded using CD spectrometer (J-815, JASCO) under the flow of nitrogen.

S.1.14. FT-IR spectroscopy. 1 mM peptide solution was prepared for FT-IR spectroscopy. The solution was measured using L1600300 Spectrum TWO LiTa FT-IR spectrometer by PerkinElmer.

S.1.15. Cell culture. HeLa, 4T1, and NIH/3T3 were supplied from Korean Cell Line Bank. Dulbecco's Modified Eagle's medium (DMEM) supplemented with 10% fetal bovine serum (FBS), 100 U/mL penicillin and 100 μg/mL streptomycin was used for HeLa, NIH/3T3 cellular growing, RPMI-1640 with 10% fetal bovine serum (FBS), 100 U/mL penicillin and 100 μg/mL streptomycin was used for 4T1. All cell lines were incubated at 37°C in a humidified atmosphere of 5% CO_2.

S.1.16. MTT assay. HeLa was seeded in 96-well plate with the density of 10,000 per well. After growing for 24 h, **Py-FFK-Az**, **Py-FFK-Biotin**, **Py-GGK-Az**, **Py-GGK-Biotin** was treated with different concentrations. After 24 h incubations, media was replaced with MTT solution. After 4 h incubation, MTT formazan was solubilized by SDS solution, and monitored using SpectraMax M5e Multi Mode Microplate Reader by Molecular Devices.

S.1.17. Flow cytometry analysis for POI uptake. HeLa and NIH/3T3 were seeded in 96-well plate with the density of 20,000 per well. 400 nM streptavidin-FITC diluted in DMEM was co-incubated with the self-assembled peptides in different concentrations. After that, the cells were washed and analyzed using FACSverse (Becton Dickinson And Company).

S.1.18. Confocal laser scanning microscopy study. HeLa and NIH/3T3 were seeded in 8-well cover glass (Lab Tek II, Thermo Scientific) with the density of 20,000 per well. 400 nM streptavidin-FITC diluted in DMEM was co-incubated with self-assembled peptides in different concentrations. After that, the cells were washed and analyzed using confocal laser scanning microscopy (LSM780 and 880 by ZEISS).

S.1.19. Endocytosis pathway analysis. HeLa was seeded in 96-well plate with the density of 20,000 per well. Then, 400 mM Sucrose, 10 mM M-β-CD, 30 μM EIPA were prepared in DMEM. After that, endocytosis inhibitor was pre-incubated toward HeLa. After 30 min, 100

μM **Py-FFK-Az** and 5μM **Py-FFK-Biotin** was co-incubated with 400 nM streptavidin-FITC. After 3 h, it was replaced with fresh media. After that, flow cytometry was conducted using FACSverse (Becton Dickinson And Company).

S.1.20. Western blot analysis for PD-L1 degradation. HeLa was seeded in 6-well plate with the density of 300,000 per well. After 24 h growing, each peptides were treated for 24 h. Then, medium were removed and cell was washed with 1X PBS. Cell line was harvested using Trypsin-EDTA. The harvested cell was further washed with 1X PBS and resuspended into 100 μL of RIPA buffer containing protease inhibitor cocktail (sigma Aldrich, P8849) at ice for 45 min. Cell lysis supernatant was collected by spin-down with 13,000 rpm for 20 min and total protein concentration was assayed using Bradford reagents. Protein was separated by SDS-PAGE and was transferred into a polyvinylidene difluoride (PVDF) membrane. PVDF membrane was blocked by 5% skim milk solution in TBS-T buffer for 1 h. After blocking step, a primary antibody (anti-PD-L1 antibody, abcam, ab213524) was incubated at 4°C during overnight. After 3 times wash with TBS-T buffer for 15 min, a secondary antibody labeled with horseradish peroxidase (HRP) was incubated at RT for 1 h. After 3 times wash with TBS-T buffer for 15 min, protein was visualized by BCIP®/NBT solution and observed by ChemiDoc Imaging Systems.

S.1.21. Immunofluorescence analysis. HeLa was seeded in 8-well cover glass (Lab Tek II, Thermo Scientific) with the density of 20,000 per well overnight. After that, different concentrations of each peptide were treated for 24 h. Then, the cells were washed using 1X PBS and were fixed with 4% paraformaldehyde for 20 min at RT, as followed by permeabilization with 0.1% Triton X-100 for 10 min at RT. Then, the cells were blocked using 10% BSA for 1 h at RT. Then, the cells were stained with primary antibody (Anti-PD-L1 antibody [EPR19759], ab213524, 1:200) at 4°C for overnight. After washing using 1X PBS-T three times, secondary antibodies (Goat Anti-Rabbit IgG H&L conjugated with Alexa Fluor® 488, ab150077, 1:1000) was treated at RT for about 1 h. DAPI (D1306, invitrogen) was used as counterstaining agent. IF signals were analyzed using confocal laser scanning microscopy (LSM780 and 880 by ZEISS).

S.1.22. Mouse model study. All animal experiments were conducted following the protocols approved by the IACUC-UNIST. BALB/c mice was purchased from Orient Bio, Korea, To produce a tumor xenograft model, 4T1 cells (0.1 mL/mice, 2 × 10^6 cells/100 μL) were subcutaneously injected into the right flank area of each mouse. Tumor growth was monitored using the digital caliper. We calculated the tumor volume using the formula [volume = (tumor length) × (tumor width)^2/2]. When the tumor volume reached 80 mm^3, four representative mice groups (i) PBS, (ii) **Py-FFK-Az**, (iii) **Py-FFK-BMS**, and (iv) 1:1 mixture for **Py-FFK-Az** and **Py-FFK-BMS** with $n = 4$ were selected. Mice were treated with 2.5 mg/kg every peptide by intratumoral injection up to 14 days. The tumor size and body weight were measured and compared.

S.1.23. Statistical analysis. All the data are expressed as mean ± standard deviation (SD). For hypothesis test, Student *t*-test was used for comparisons of two groups. For the comparison of multiple groups, ANOVA test was used with the Tucky post-hoc analysis. Details for statistical analyses are indicated in each figure. ***$p < 0.001$, **$p < 0.01$, and *$p < 0.05$.

S2. Supplementary Figures

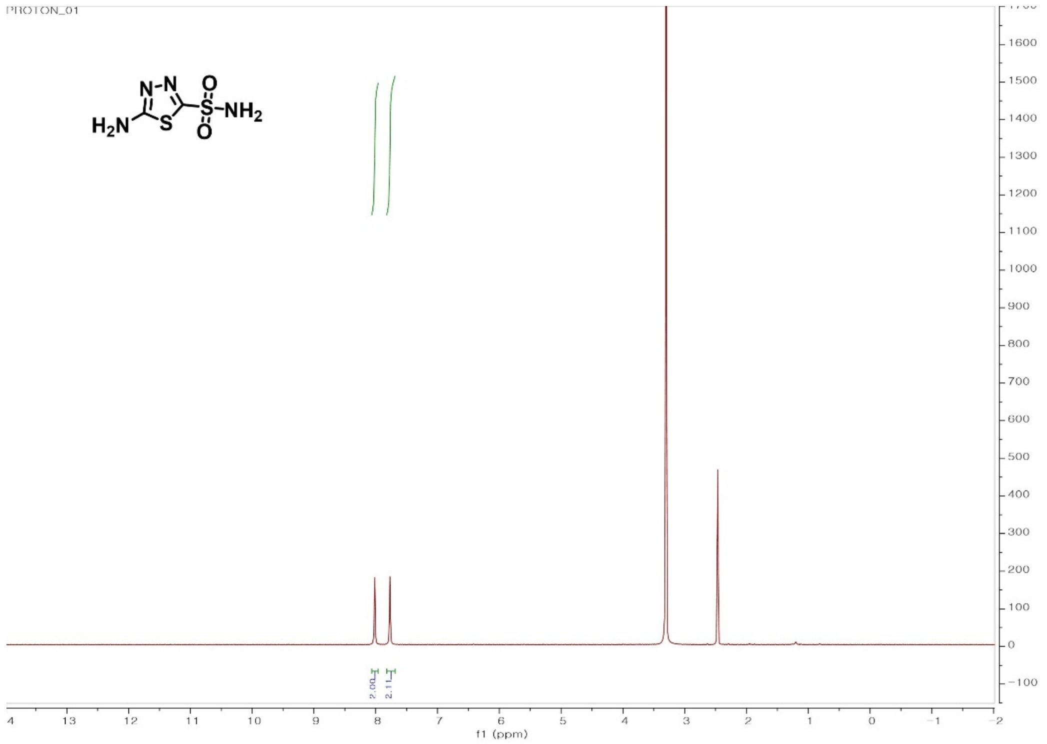

Supplementary Figure 1. ^{1}H-NMR spectrum of 5-amino-1,3,4-thiadiazole-2-sulfonamide

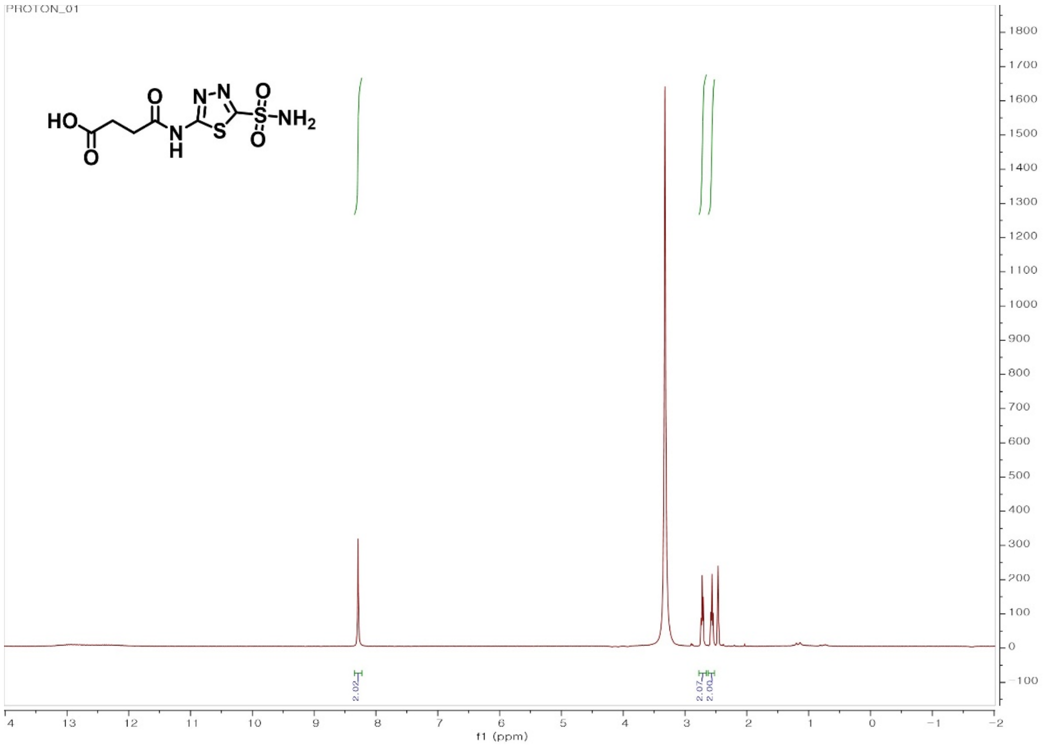

Supplementary Figure 2. ^{1}H-NMR spectrum of 4-oxo-4-((5-sulfamoyl-1,3,4-thiadiazol-2-yl)amino)butanoic acid

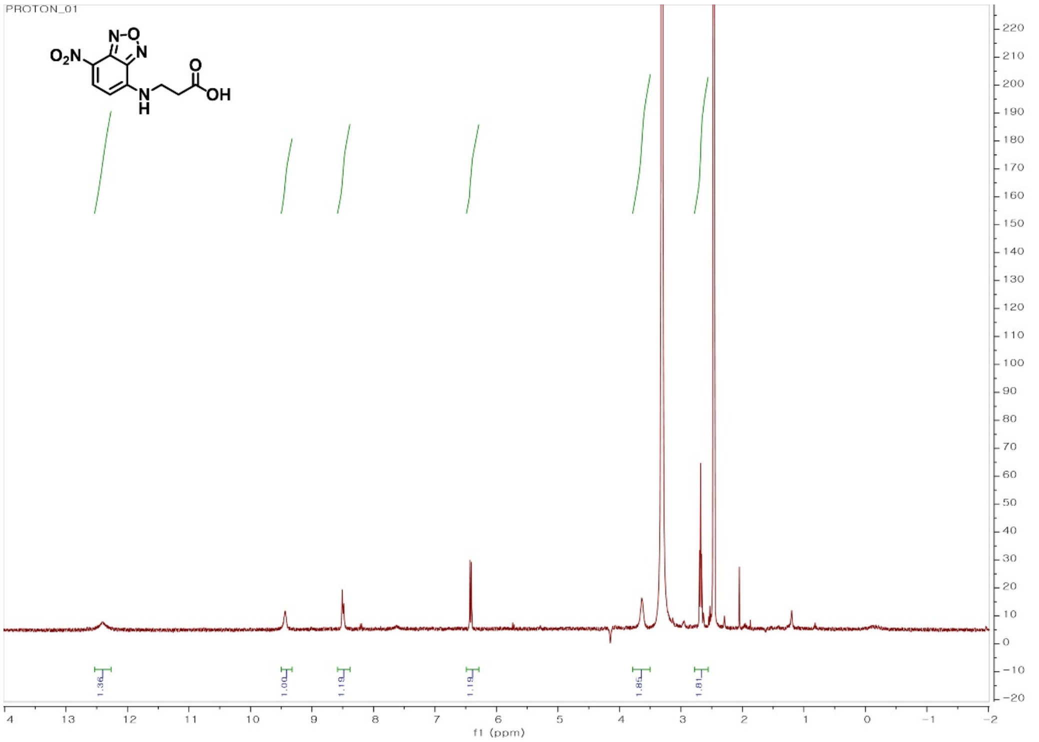

Supplementary Figure 3. ^{1}H-NMR spectrum of 3-((7-nitrobenzo[c][1,2,5]oxadiazol-4-yl)amino)propanoic acid

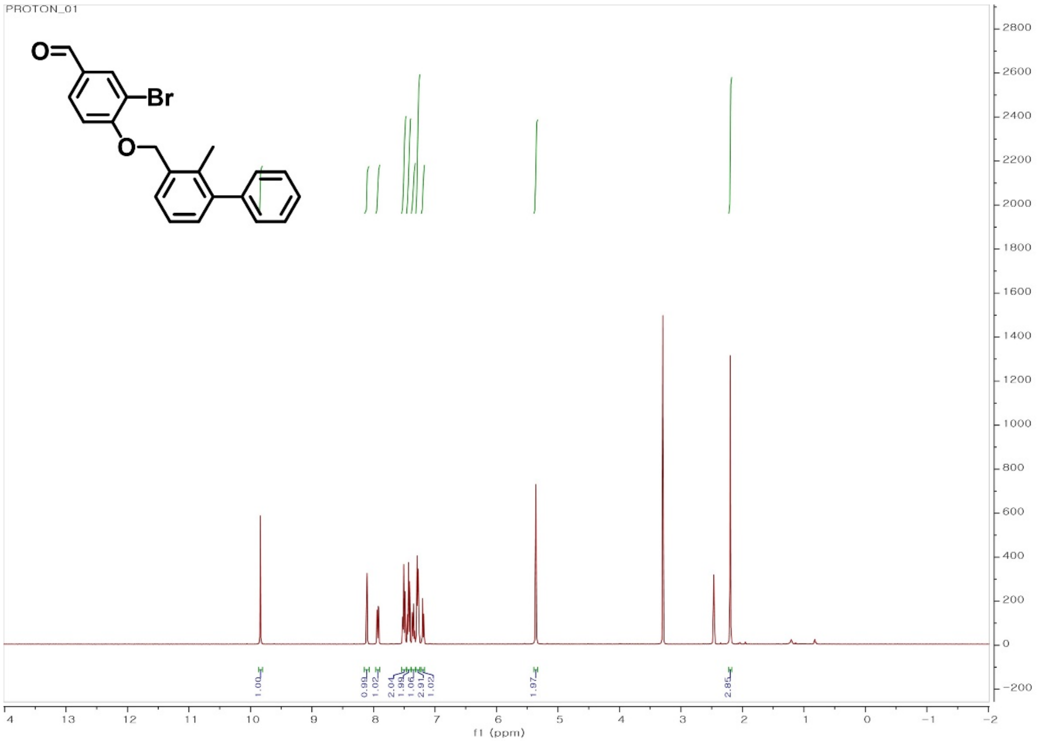

Supplementary Figure 4. ^{1}H-NMR spectrum of 3-bromo-4-((2-methyl-[1,1'-biphenyl]-3-yl)methoxy)benzaldehyde

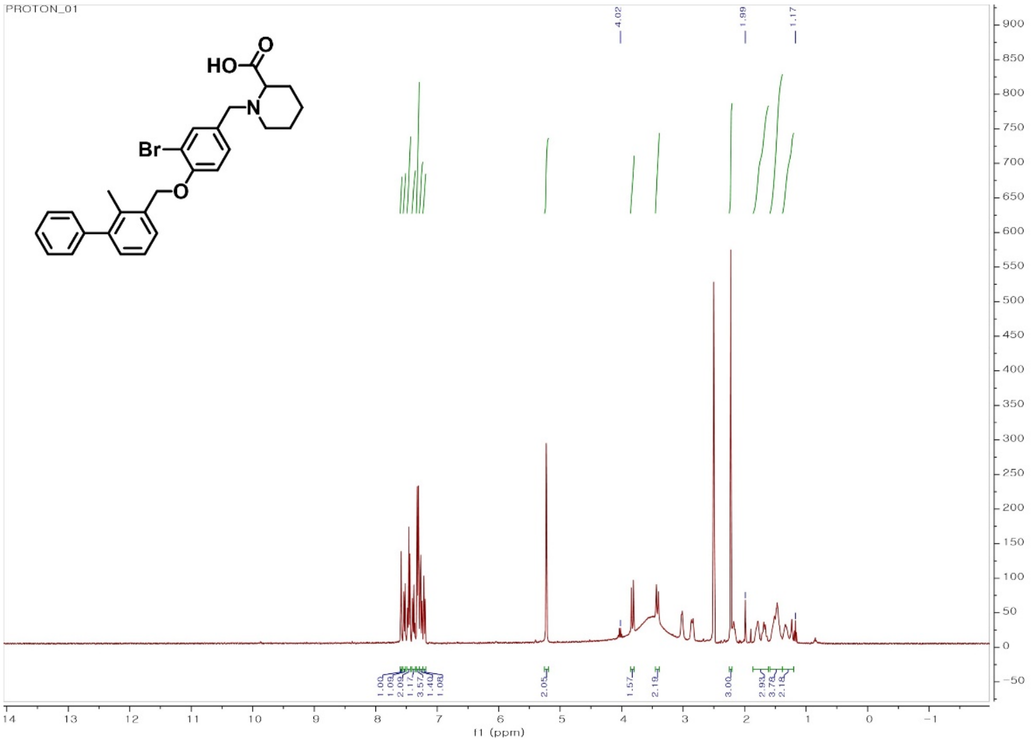

Supplementary Figure 5. ^{1}H-NMR spectrum of 1-[[3-bromo-4-[(2-methyl[1,1'-biphenyl]-3-yl)methoxy]phenyl]methyl]-2-piperidinecarboxylic acid **(BMS-8)**

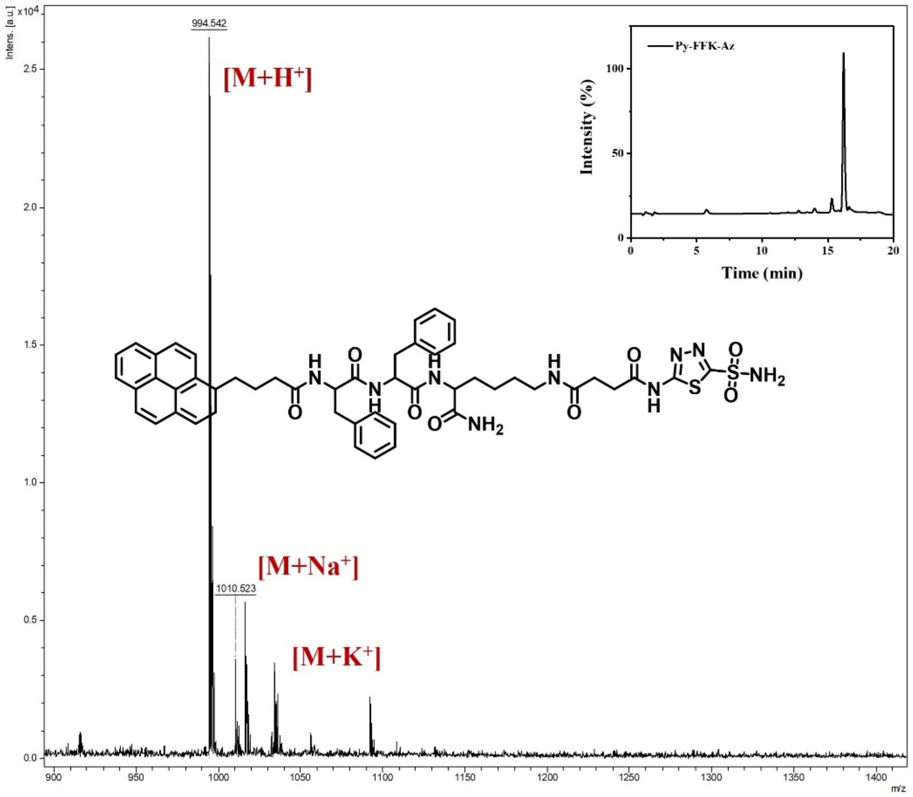

Supplementary Figure 6. MALDI-TOF/TOF for **Py-FFK-Az**. Inset : Analytical HPLC trace for **Py-FFK-Az**.

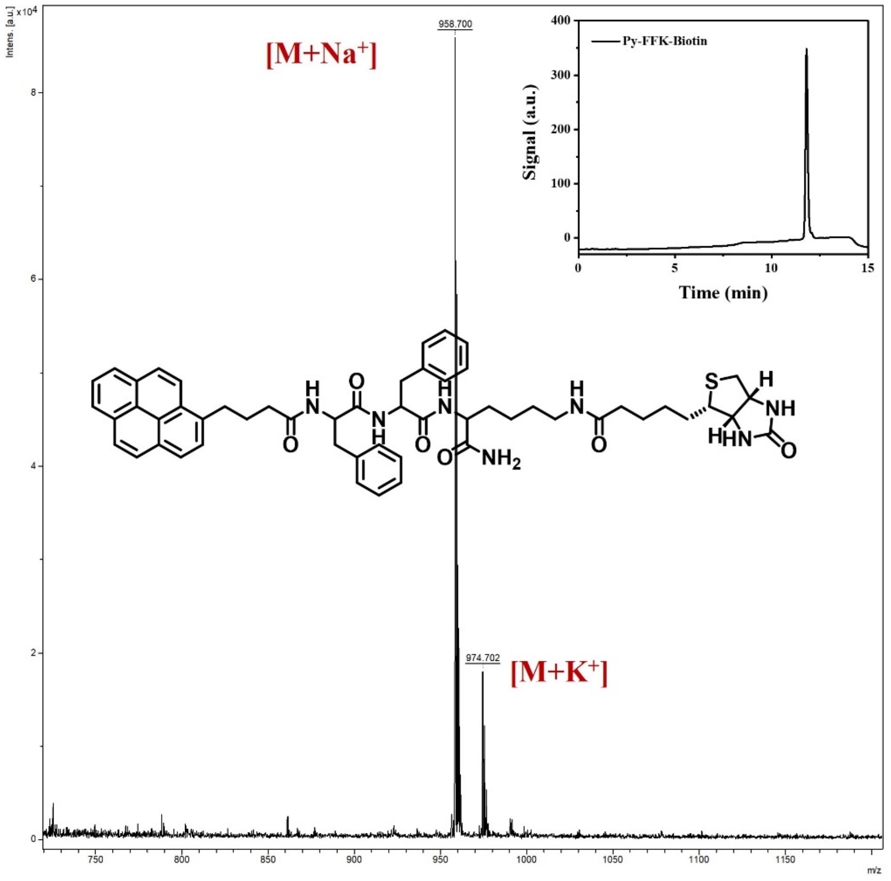

Supplementary Figure 7. MALDI-TOF/TOF for **Py-FFK-Biotin**. Inset : Analytical HPLC trace for **Py-FFK-Biotin**.

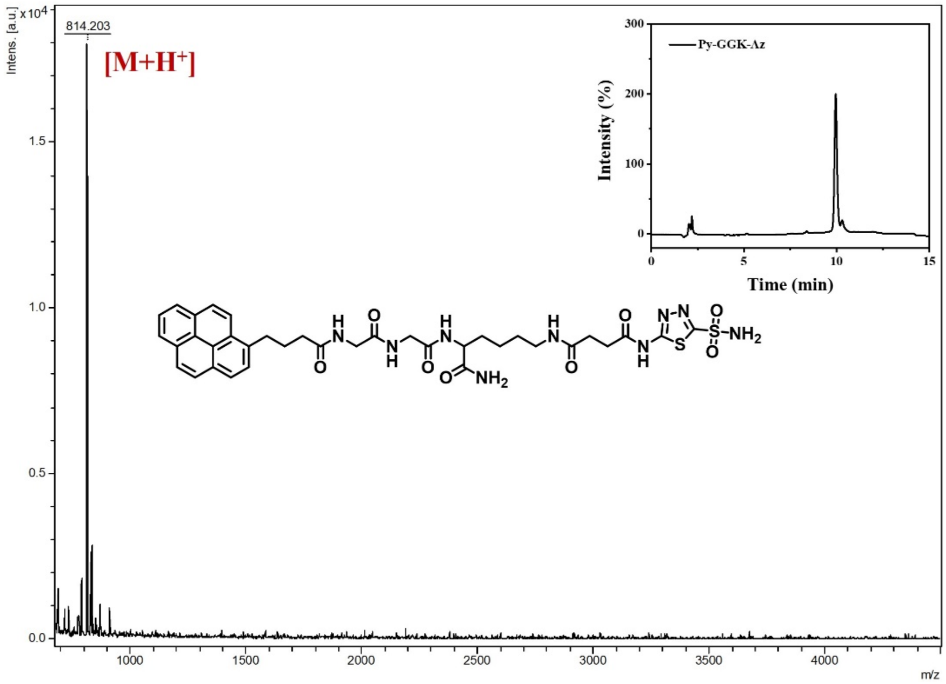

Supplementary Figure 8. MALDI-TOF/TOF for **Py-GGK-Az**. Inset : Analytical HPLC trace for **Py-GGK-Az**.

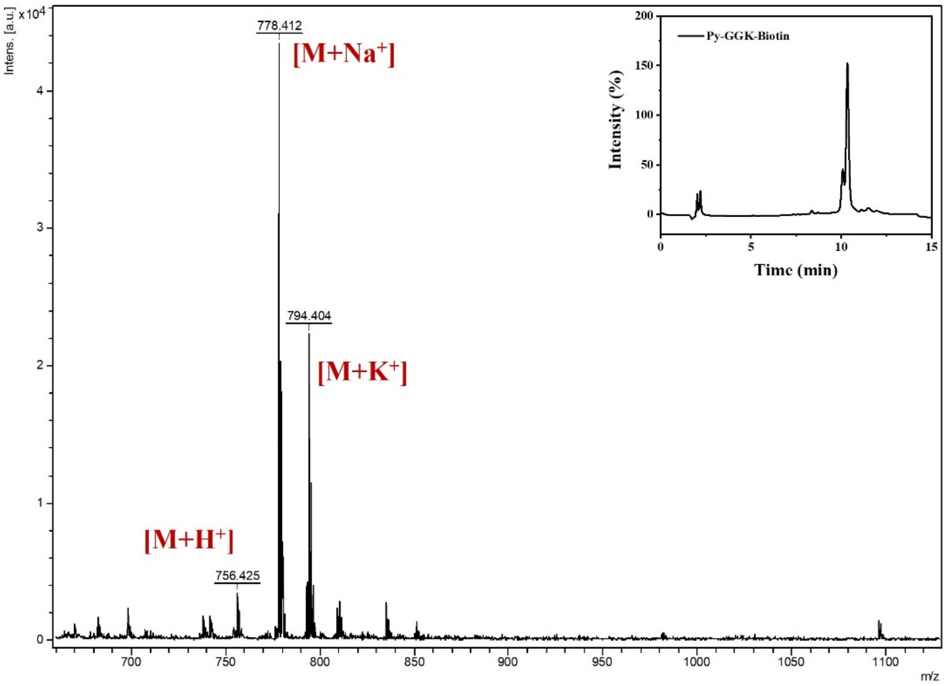

Supplementary Figure 9. MALDI-TOF/TOF for **Py-GGK-Biotin**. Inset : Analytical HPLC trace for **Py-GGK-Biotin**.

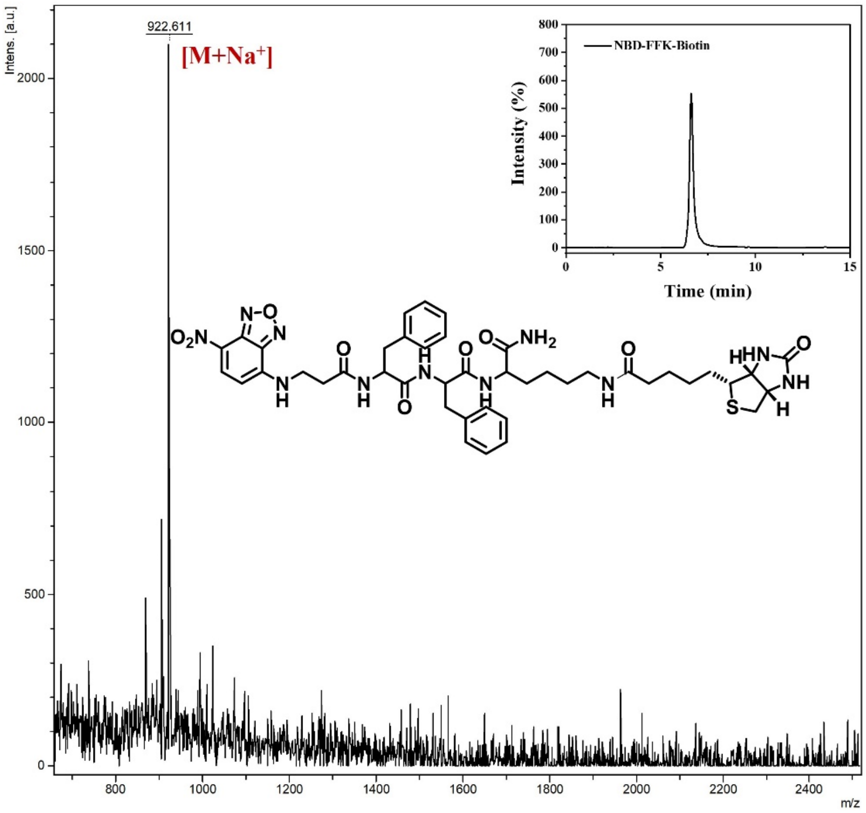

Supplementary Figure 10. MALDI-TOF/TOF for **NBD-FFK-Biotin**. Inset : Analytical HPLC trace for **NBD-FFK-Biotin**.

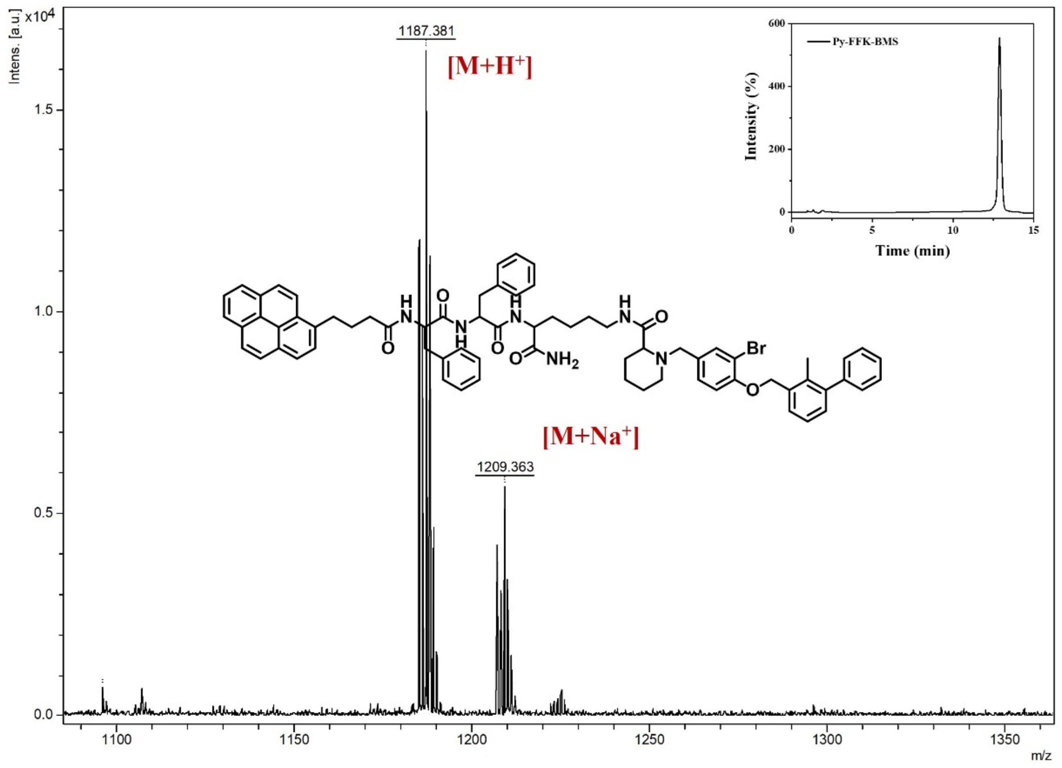

Supplementary Figure 11. MALDI-TOF/TOF for **Py-FFK-BMS**. Inset : Analytical HPLC trace for **Py-FFK-BMS**.

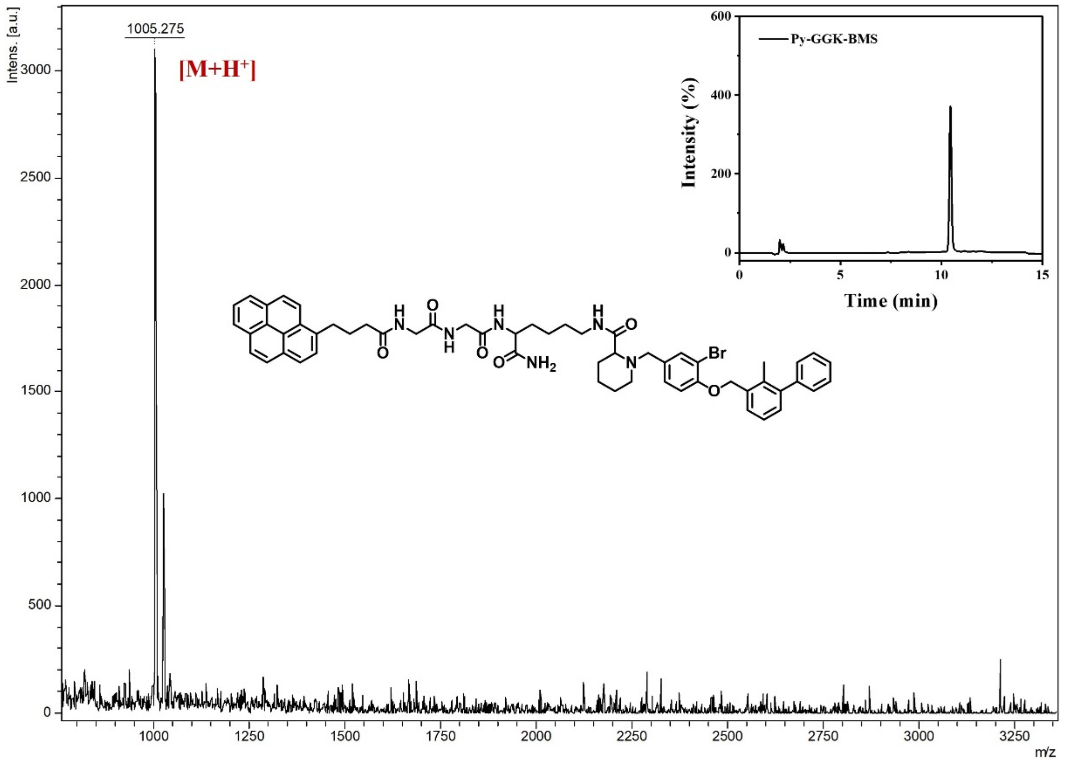

Supplementary Figure 12. MALDI-TOF/TOF for **Py-GGK-BMS**. Inset : Analytical HPLC trace for **Py-GGK-BMS**.

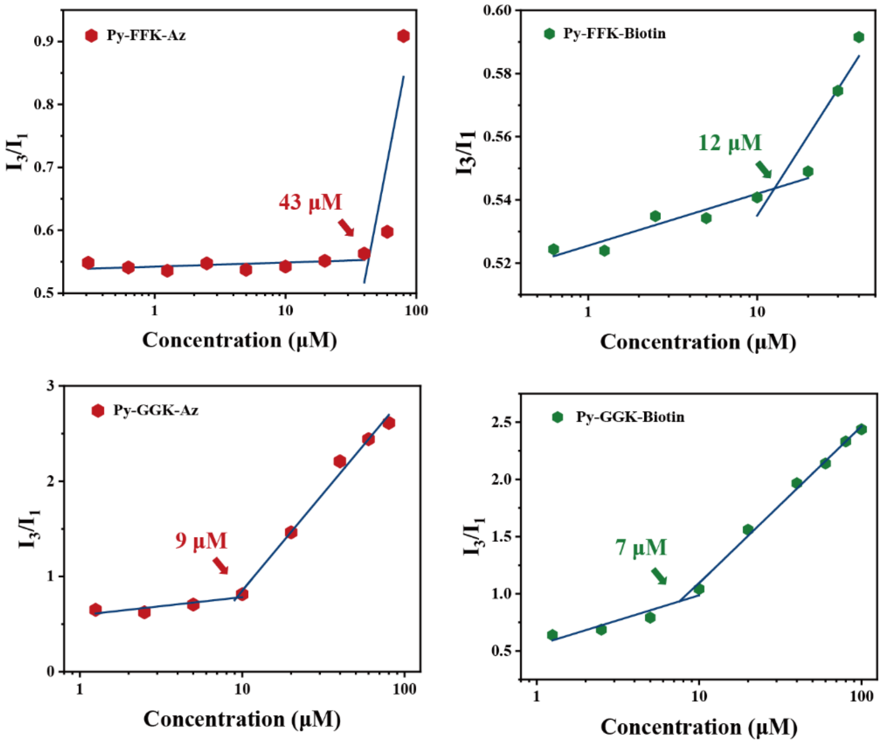

Supplementary Figure 13. CAC measurement toward **Py-FFK-Az**, **Py-FFK-Biotin**, **Py-GGK-Az**, and **Py-GGK-Biotin** using pyrene emission method. The arrow indicates CAC value of each peptide.

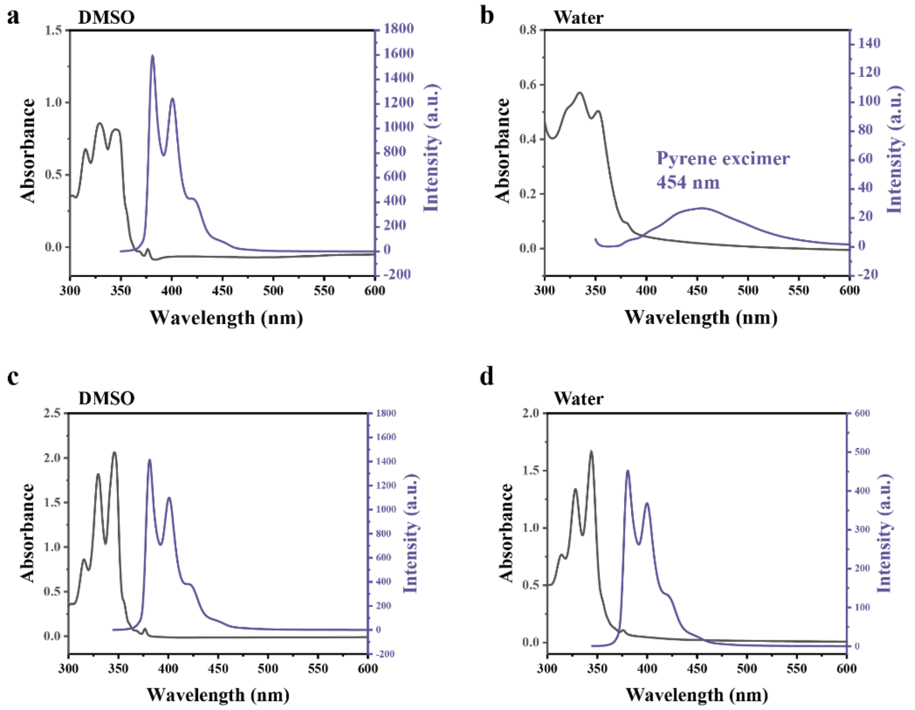

Supplementary Figure 14. a-b) UV-Vis spectrum for **Py-FFK-Az** (100 µM) in a) DMSO and b) aqueous solution; c-d) UV-Vis spectrum for **Py-GGK-Az** (100 µM) in c) DMSO and d) aqueous solution

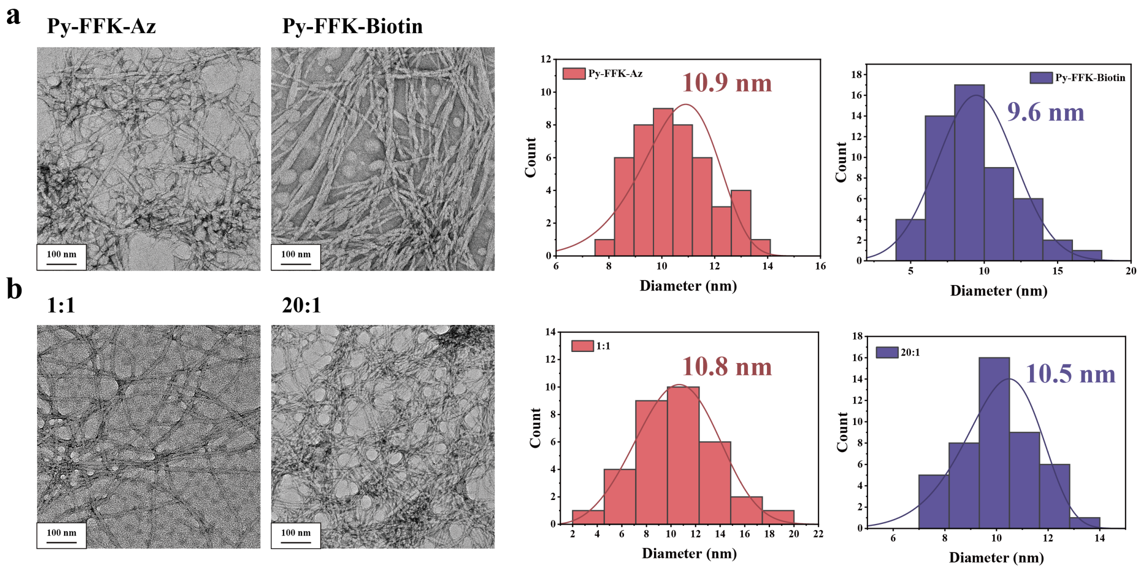

Supplementary Figure 15. a-b) Magnified TEM image a) for **Figure 1a** and b) for **Figure 1c**. Fiber diameter was analyzed by histogram using image J software.

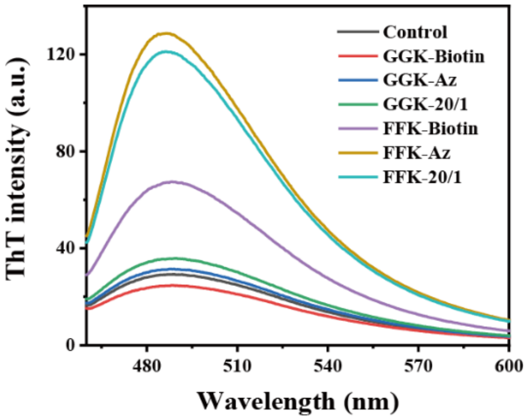

Supplementary Figure 16. Fluorescence intensity for ThT inside self-assembly nanostructure was measured.

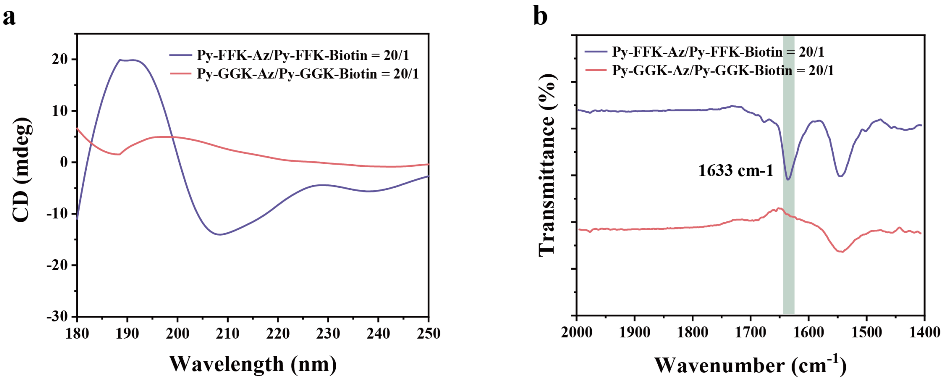

Supplementary Figure 17. a) Circular dichroism spectrum for the mixture of FFK- and GGK-based peptides; b) FT-IR spectrum for the mixture of FFK- and GGK-based peptides. Amide I band at 1633 cm^{-1} indicates the parallel beta-sheet structure.

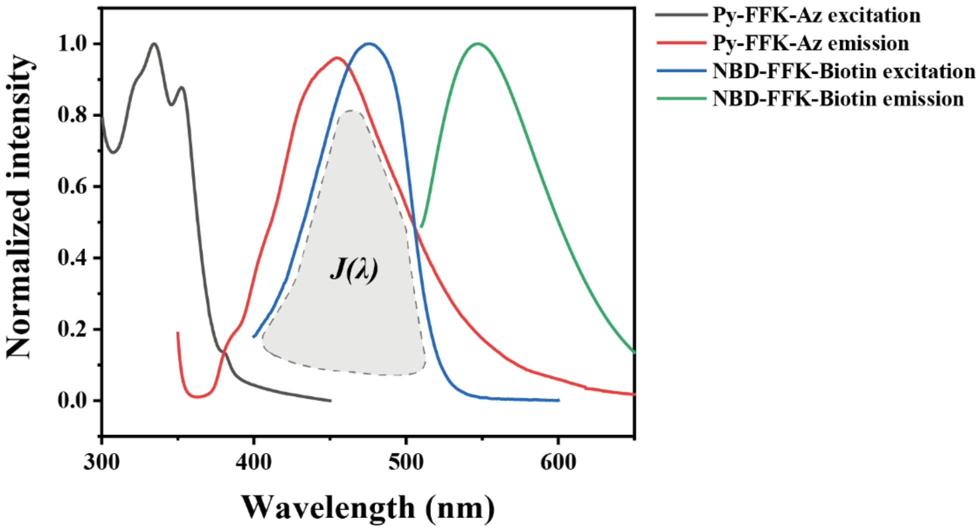

Supplementary Figure 18. The excitation and emission spectrum for **Py-FFK-Az** (100 μM) and **NBD-FFK-Biotin** (100 μM). donor emission spectrum (excimer generated from **Py-FFK-Az**) are significantly overlapped with the acceptor excitation overlap (**NBD-FFK-Biotin**).

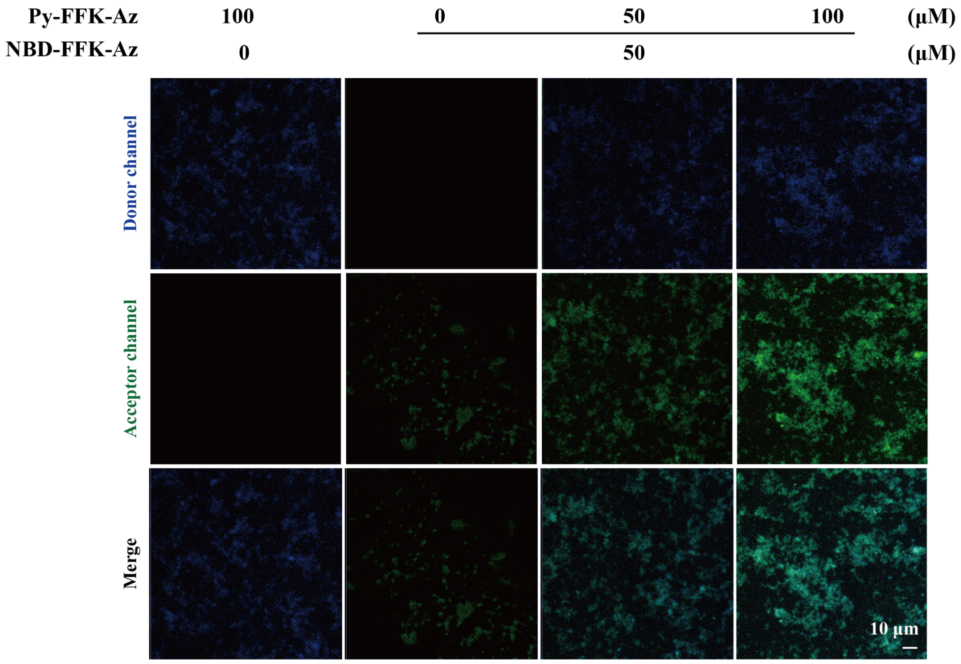

Supplementary Figure 19. CLSM image showing the FRET phenomena between two probes. The excitation laser was set to 405 nm. The emission spectrum band for the donor channel (excimer peak form **Py-FFK-Az**) was set to 454 nm, and the emission spectrum band for acceptor channel (NBD peak from **NBD-FFK-Biotin**) was set to 550 nm.

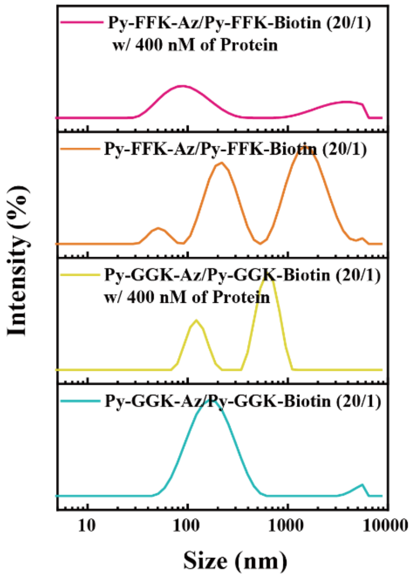

Supplementary Figure 20. Acetazolamide conjugated peptides (**Py-FFK-Az** and **Py-GGK-Az**) and biotin conjugated peptides (**Py-FFK-Biotin** and **Py-GGK-Biotin**) were mixed in the ratio of 20 to 1, and intensity profile with or without streptavidin (400 nM) was measured using DLS.

a

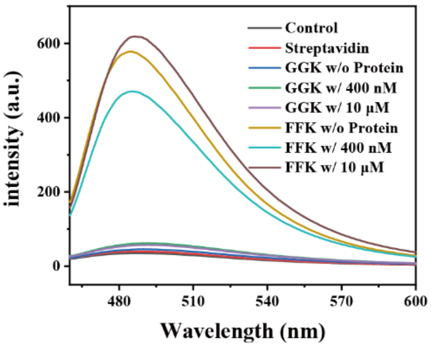

b

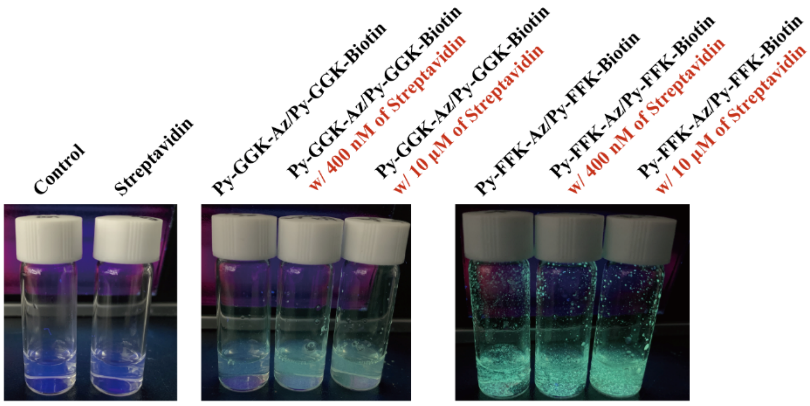

Supplementary Figure 21. a) Fluorescence measurements for ThT encapsulated in each peptide with or without streptavidin. Acetazolamide conjugated peptides (**Py-FFK-Az** and **Py-GGK-Az**) and biotin conjugated peptides (**Py-FFK-Biotin** and **Py-GGK-Biotin**) were mixed in the ratio of 20 to 1; b) Optical image for ThT fluorescence under 365 nm irradiation. Data are presented as mean ± SD (*n* = 3)

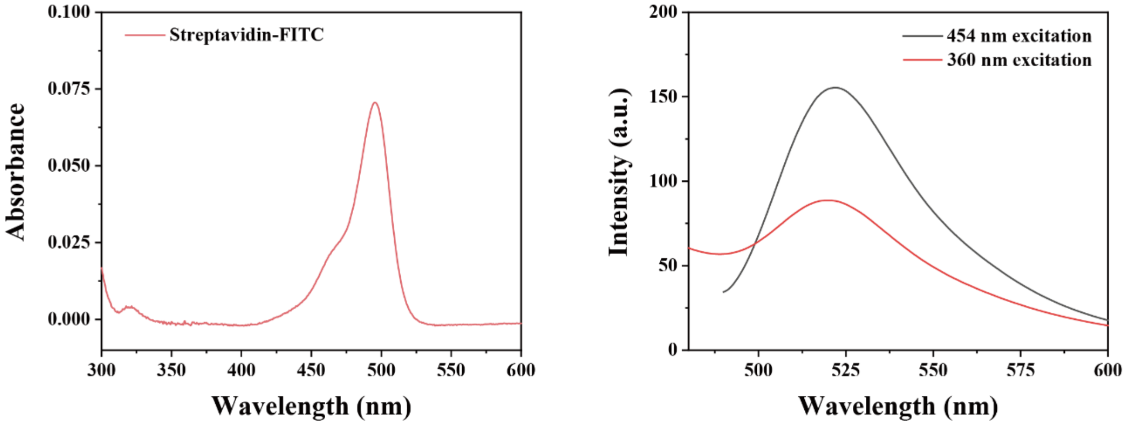

Supplementary Figure 22. UV/Vis absorbance and fluorescence intensity measurement at 360 nm and 454 nm excitation for streptavidin-FITC

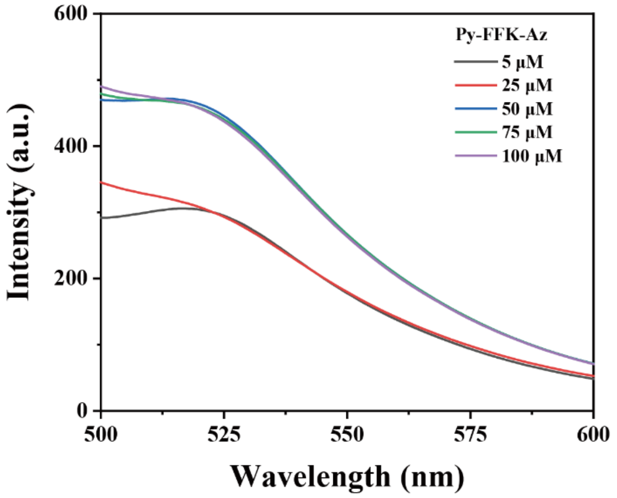

Supplementary Figure 23. FRET signal measurement in **Py-FFK-Az** concentration dependent manner. 5 μM Py-FFK-Biotin with 400 nM streptavidin-FITC was co-incubated.

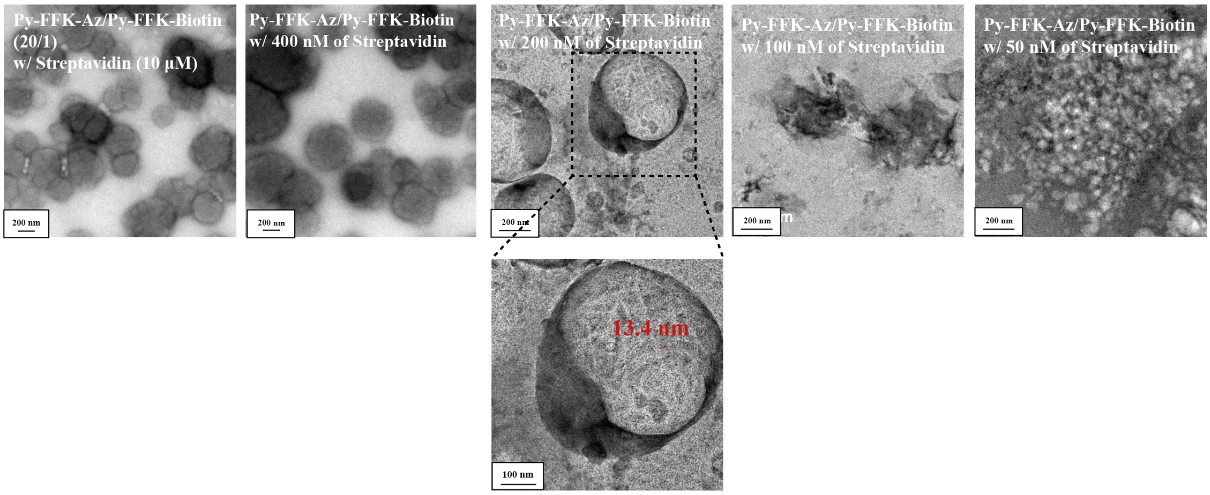

Supplementary Figure 24. TEM analysis for **Py-FFK-Az/Py-FFK-Biotin** (20/1 ratio) at varying concentration of streptavidin.

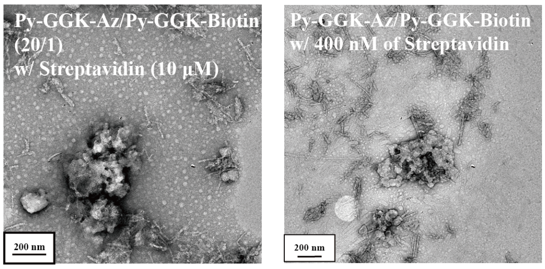

Supplementary Figure 25. TEM analysis for **Py-GGK-Az/Py-GGK-Biotin** (20/1 ratio) at varying concentration of streptavidin.

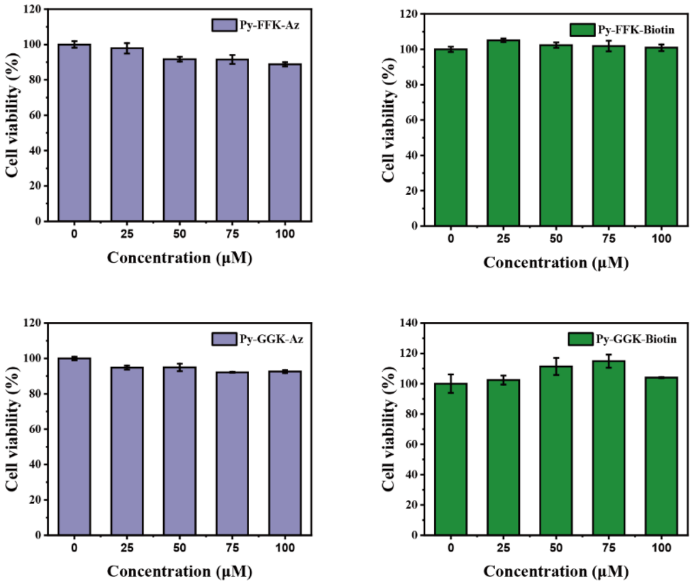

Supplementary Figure 26. Cell viability measurement toward HeLa for **Py-FFK-Az**, **Py-FFK-Biotin**, **Py-GGK-Az**, and **Py-GGK-Biotin** using MTT assay. Data are presented as mean ± SD (n = 3)

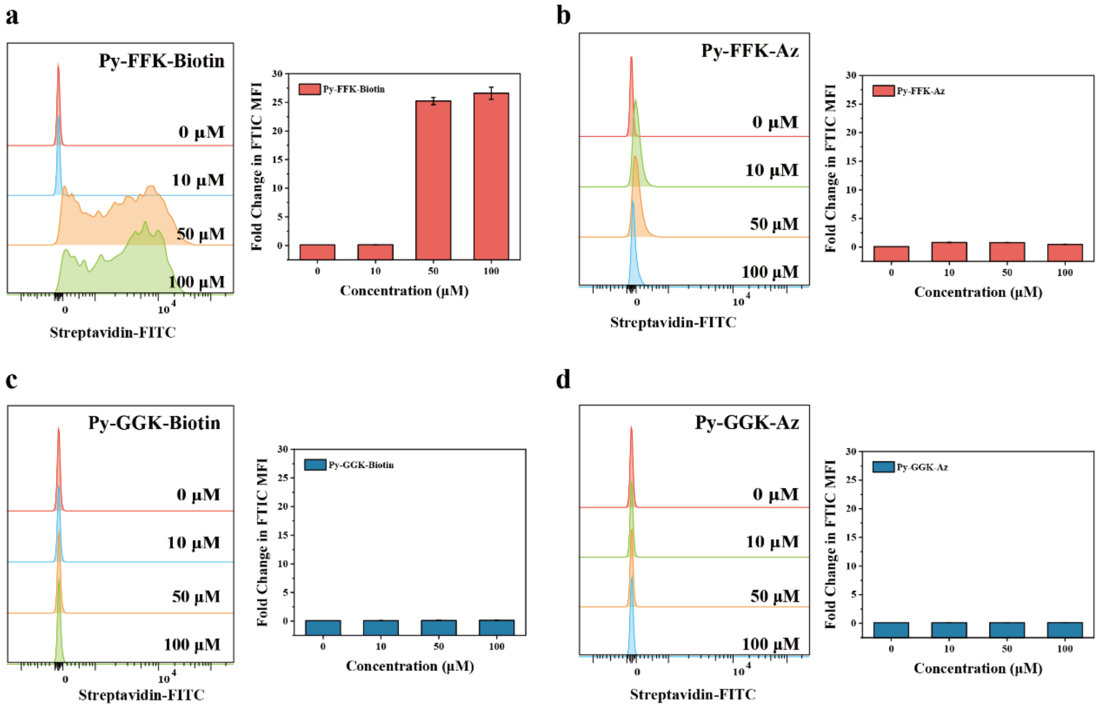

Supplementary Figure 27. a-d) Streptavidin-FITC uptake measurement using flow cytometry with the incubation of each synthesized peptide in concentration dependent manner toward HeLa.

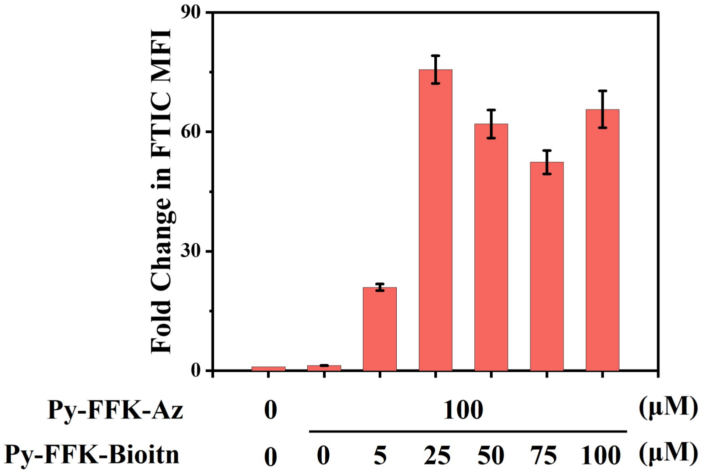

Supplementary Figure 28. Fold change in streptavidin-FITC MFI in **Py-FFK-Biotin** concentration dependent manner with 100 μM **Py-FFK-Az** treatment toward HeLa. It was incubated for 4 h. Data are presented as mean ± SD (n=3).

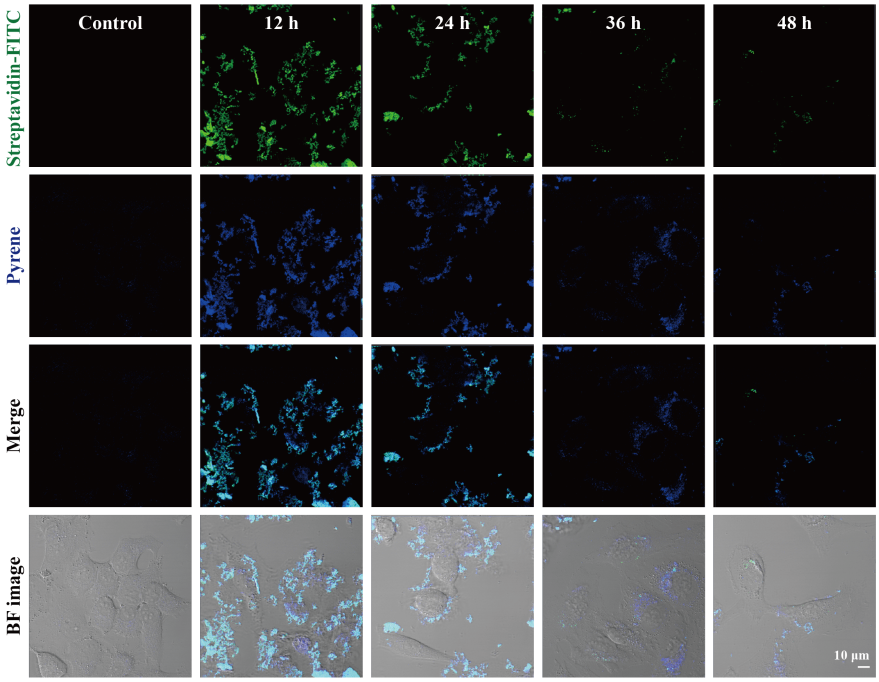

Supplementary Figure 29. CLSM image showing the fluorescence from streptavidin-FITC and Supra-LYTAC and its merge image up to 48 h incubation.

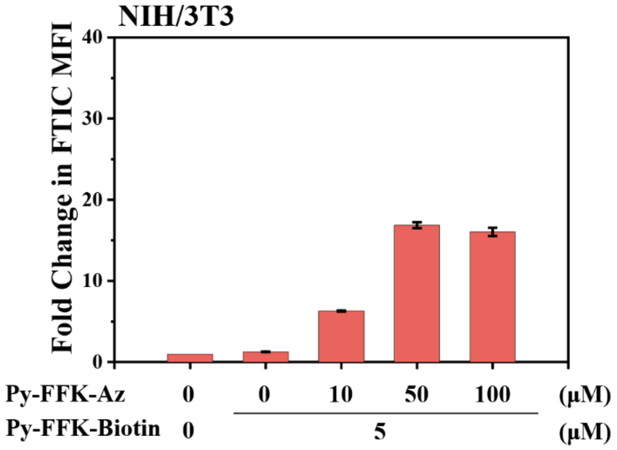

Supplementary Figure 30. Fold change in streptavidin-FITC MFI for FFK-based peptides in **Py-FFK-Az** concentration dependent manner toward NIH/3T3. Data are presented as mean ± SD (n = 3)

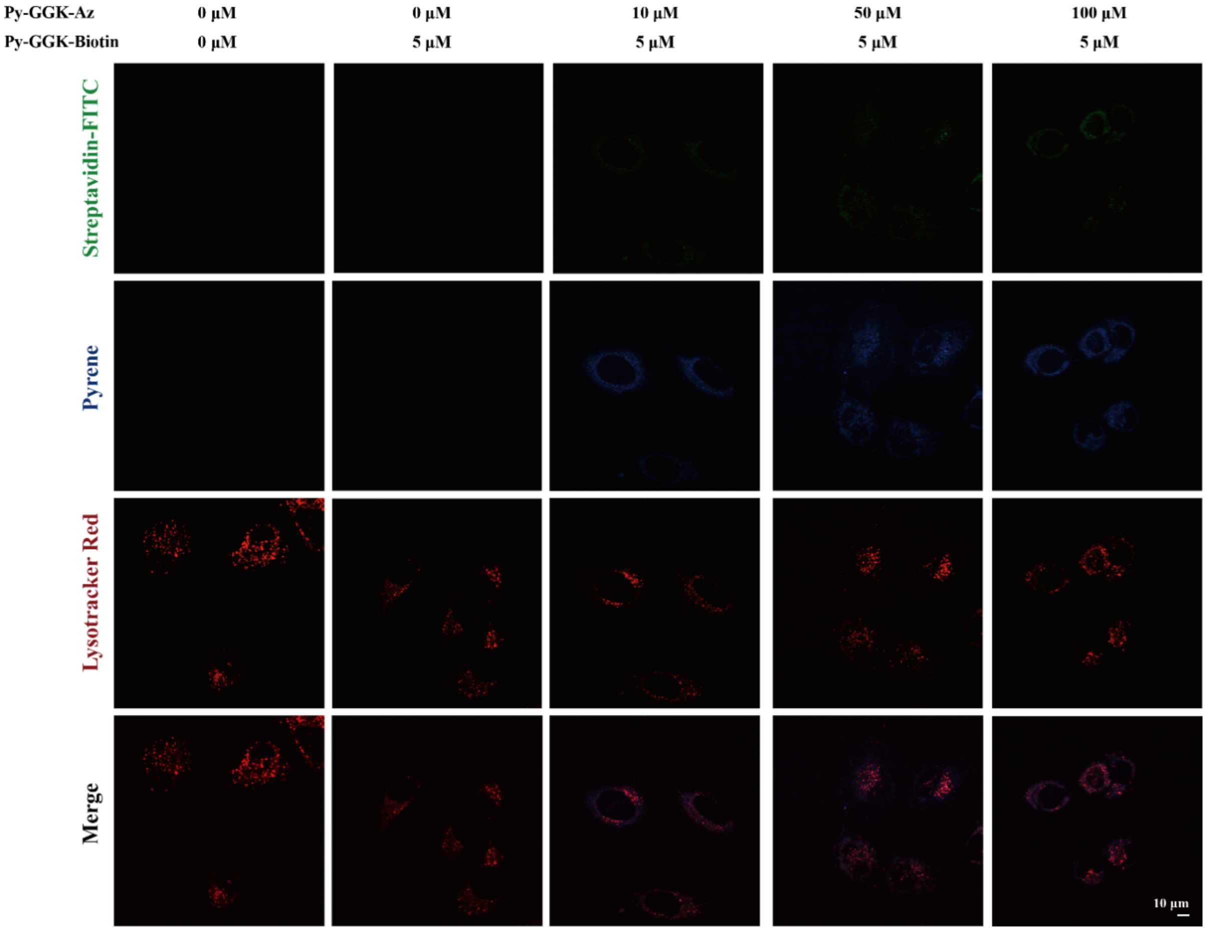

Supplementary Figure 31. CLSM image showing streptavidin-FITC internalization in concentration dependent manner for GGK-based peptide toward HeLa.

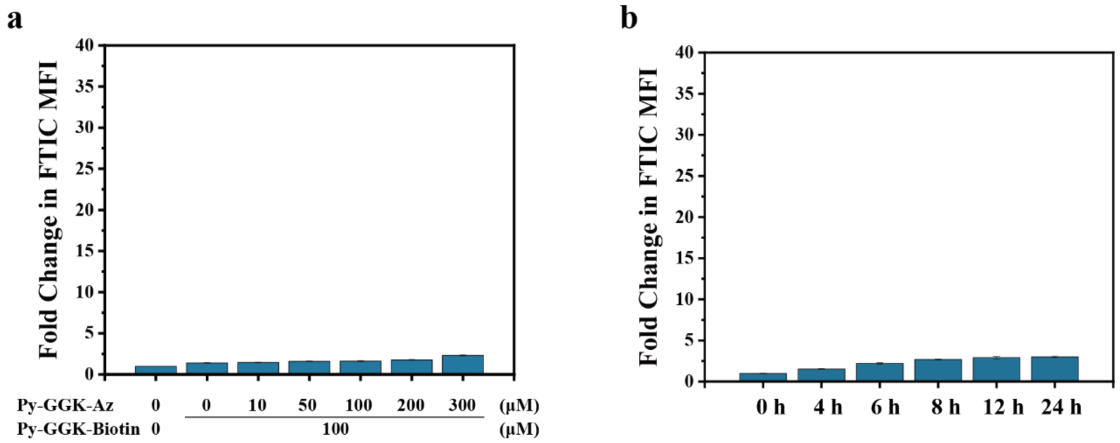

Supplementary Figure 32. a) Fold change in streptavidin-FITC MFI for GGK-based peptides in **Py-GGK-Az** concentration dependent manner toward HeLa. b) Fold change in streptavidin-FITC MFI with incubation of 300 μM **Py-GGK-Az** and 100 μM **Py-GGK-Biotin** in time dependent manner toward HeLa. Data are presented as mean ± SD (n = 3)

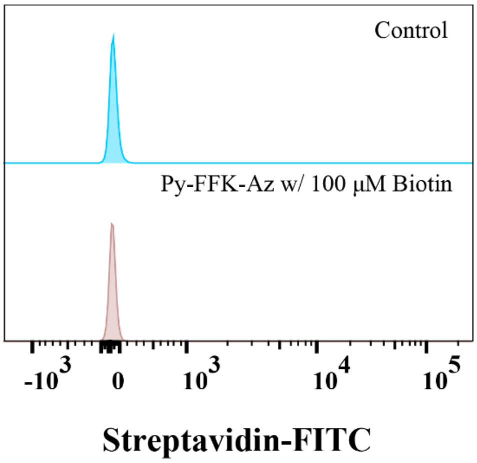

Supplementary Figure 33. Flow cytometry analysis for streptavidin-FITC uptake toward HeLa after incubation 100 μM **Py-FFK-Az** and **biotin**.

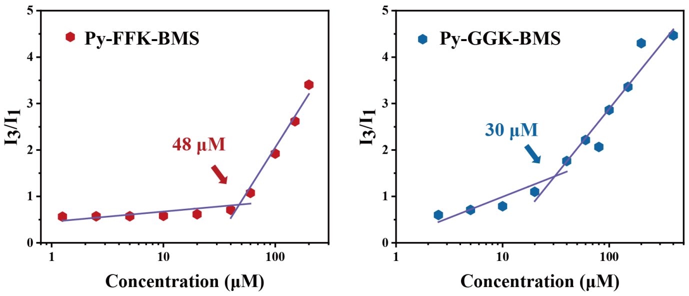

Supplementary Figure 34. CAC measurement toward **Py-FFK-BMS** and **Py-GGK-BMS** using pyrene emission method. The arrow indicates CAC value of each peptide.

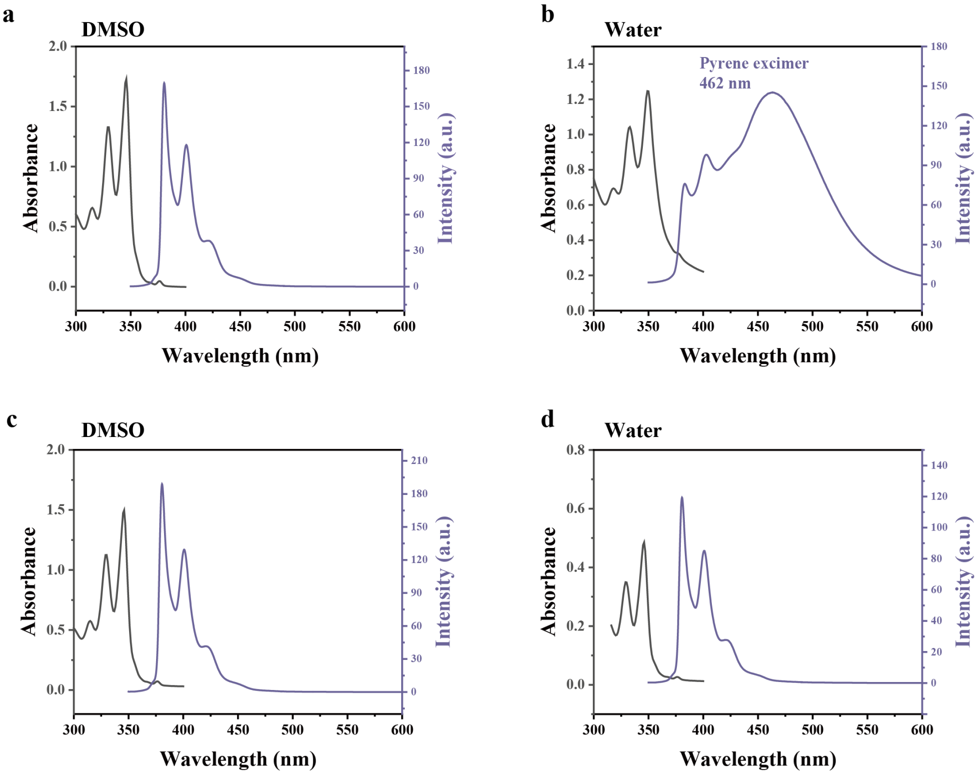

Supplementary Figure 35. a-b) UV-vis and fluorescence spectrum for **Py-FFK-BMS** (100 μM) in a) DMSO and b) aqueous solution; c-d) UV-vis and fluorescence spectrum for **Py-GGK-BMS** (100 μM) in c) DMSO and d) aqueous solution.

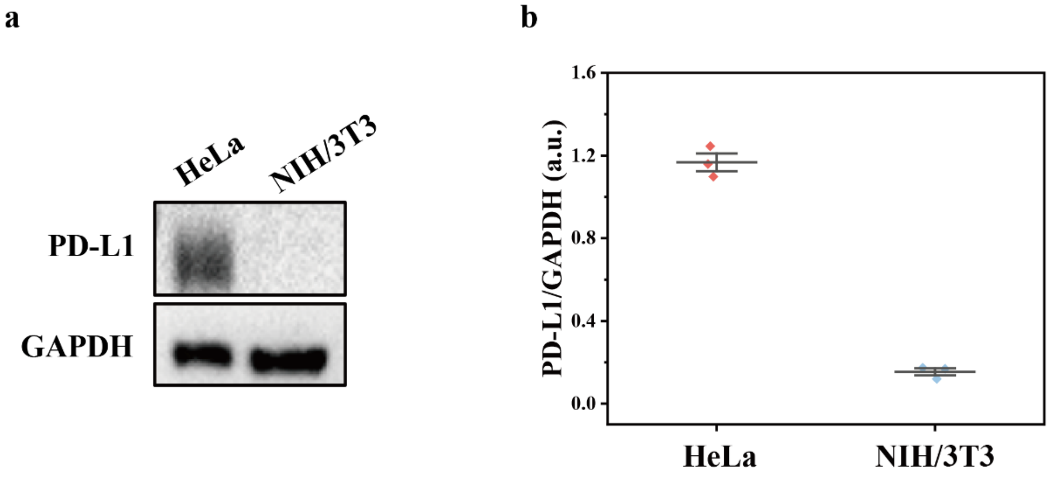

Supplementary Figure 36. a) Western blot image showing expression level for PD-L1 in HeLa and NIH/3T3. b) The intensity for PD-L1/GAPDH for HeLa and NIH/3T3 was plotted. Data are presented as mean ± SD (n = 3)

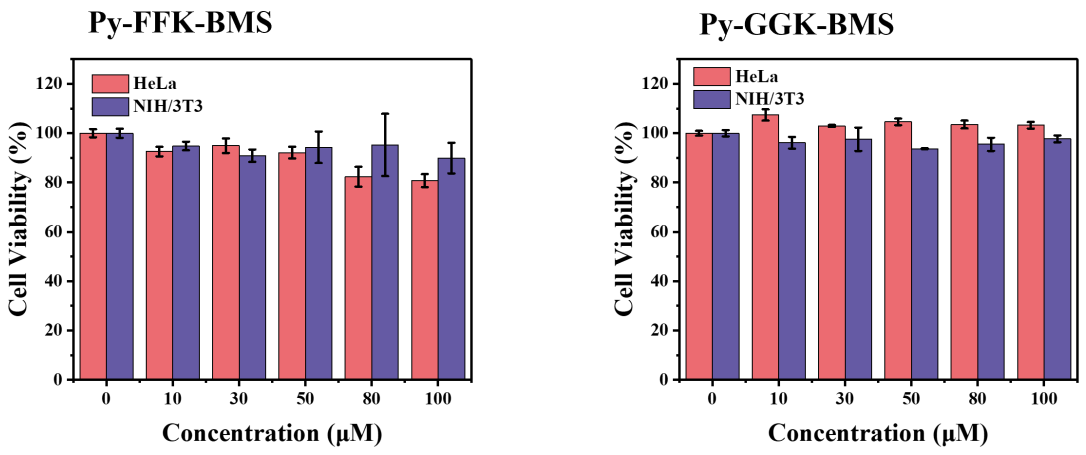

Supplementary Figure 37. Cell viability measurement toward HeLa and NIH/3T3 for **Py-FFK-BMS** and **Py-GGK-BMS** by MTT assay. Data are presented as mean ± SD (n = 3)

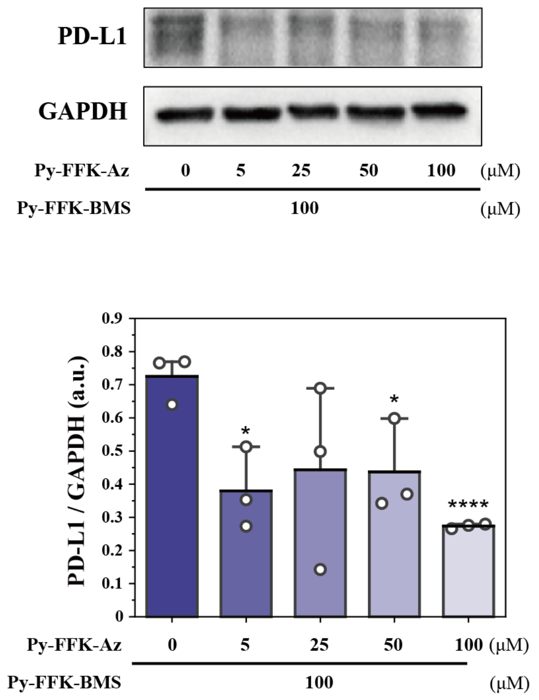

Supplementary Figure 38. WB analysis for PD-L1 degradation for **Py-FFK-Az** concentration dependent manner with 100 μM **Py-FFK-BMS** after 24 h incubation. Data are presented as mean ± SD. * $P < 0.05$ and ** $P < 0.01$ from student's t test.

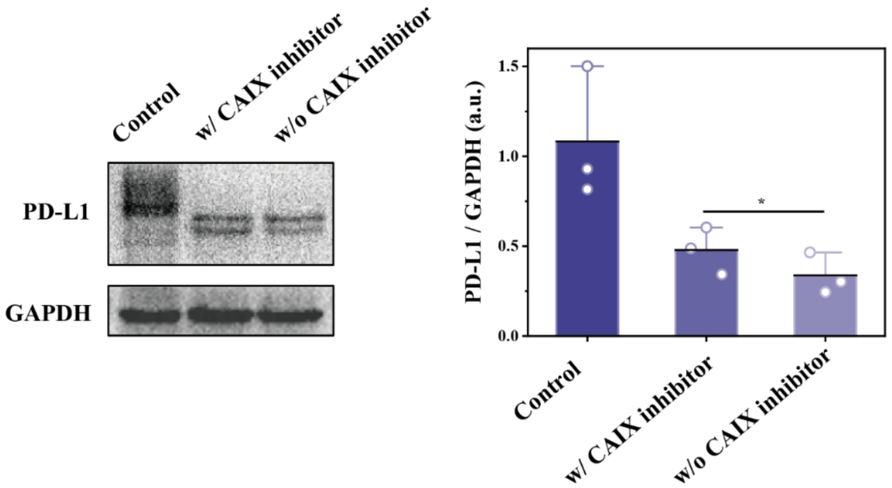

Supplementary Figure 39. a) WB analysis for PD-L1 degradation with 100 μM acetazoalmide (CAIX inhibitor) and without acetazoalmide. 100 μM **Py-FFK-Az** and **Py-FFK-BMS** were co-incubated. Data are presented as mean ± SD (n = 3). * $P < 0.05$, ** $P < 0.01$, *** $P < 0.001$, and **** $P < 0.0001$ from student's t test.

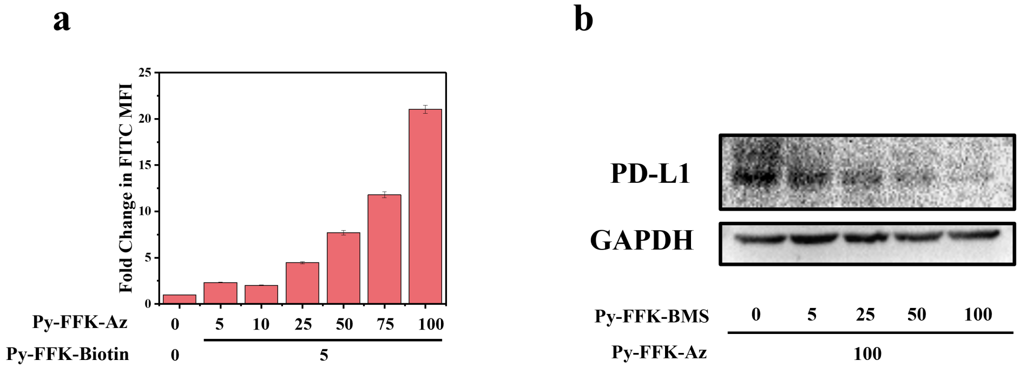

Supplementary Figure 40. a) Fold change of streptavidin-FITC with with 5 μM **Py-FFK-Biotin** in **Py-FFK-Az** concentration dependent manner after 4 h incubation toward 4T1. Data are presented as mean ± SD (n = 3); b) WB analysis for PD-L1 degradation for **Py-FFK-BMS** concentration dependent manner with 100 μM **Py-FFK-Az** after 24 h incubation toward 4T1.

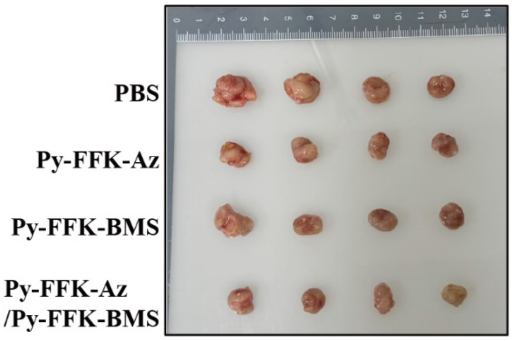

Supplementary Figure 41. Optical image showing 4T1 tumor sizes after sacrificing the mouse.

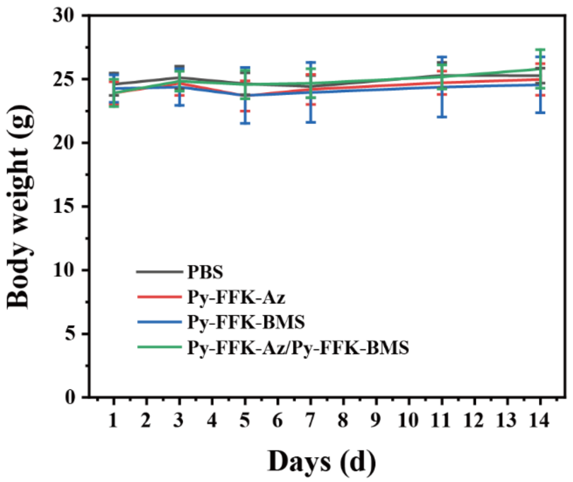

Supplementary Figure 42. Body weight measurement of every mice during treatment. Data are presented as mean ± SD (*n* = 4)

S3. References

[1] D. Kim, S. Kim, G. Park, H. Choi, and J.-H. Ryu *JACS Au*, **2022**, *2*, 2539-2547

[2] M. T. Jeena, L. Palanikumar, E. M. Go, I. Kim, M. G. Kang, S. Lee, S. Park, H. Choi, C. Kim, S.-M. Jin, S. C. Bae, H.-W. Rhee, E. Lee, S. K. Kwak, J.-H. Ryu *Nat. Commun*. **2017**, *8*, 26
